# Abundant bacterial nucleoid-associated protein H-NS limits plasmid transfer through mechanical modification of DNA

**DOI:** 10.1093/nar/gkaf928

**Published:** 2025-09-23

**Authors:** Mingyue Fei, Mengdie Fang, Qi Zhou, Ziyan Chen, Mengxin Gong, Fabai Wu, Changfu Tian, Dongchang Sun

**Affiliations:** College of Biotechnology and Bioengineering, Zhejiang University of Technology, Hangzhou, Zhejiang 310014, China; School of Laboratory Medicine and Bioengineering, Zhejiang Provincial People’s Hospital (Affiliated People’s Hospital), Hangzhou Medical College, Hangzhou, Zhejiang 310000, China; College of Biotechnology and Bioengineering, Zhejiang University of Technology, Hangzhou, Zhejiang 310014, China; College of Biotechnology and Bioengineering, Zhejiang University of Technology, Hangzhou, Zhejiang 310014, China; College of Biotechnology and Bioengineering, Zhejiang University of Technology, Hangzhou, Zhejiang 310014, China; College of Biotechnology and Bioengineering, Zhejiang University of Technology, Hangzhou, Zhejiang 310014, China; School of Life Sciences, College of Science, Eastern Institute of Technology, Ningbo, Zhejiang 315200, China; State Key Laboratory of Plant Environmental Resilience, MOA Key Laboratory of Soil Microbiology, Rhizobium Research Center, College of Biological Sciences, China Agricultural University, Beijing 100193, China; College of Biotechnology and Bioengineering, Zhejiang University of Technology, Hangzhou, Zhejiang 310014, China

## Abstract

The ongoing arms race between prokaryotes and mobile genetic elements (MGEs) gives rise to a myriad of host-defense systems that detect and degrade invading nucleic acids. However, it is yet unclear whether changing the mechanical properties of nucleic acids can impact plasmid/phage invasion. Here, we demonstrate that H-NS, an abundant nucleoid-associated protein (NAP), limits plasmid transfer by directly binding to and oligomerizing along with transforming double-stranded DNA. The constitutive defense function of H-NS can be complemented by convergently evolved NAPs from different phyla. H-NS proteins form intramolecular bridges between DNA duplexes within incoming plasmids. Different from other defense systems that exist at low levels prior to detecting MGEs, major NAPs exist in high abundance, which may moonlight as a constant plasmid surveillance agent. Our study implies that mechanical modification of nucleic acids may be an underexplored mechanism for prokaryotic immunity, which could help balance the advantages and disadvantages of MGEs.

## Graphical Abstract

**Graphical Abstract:**
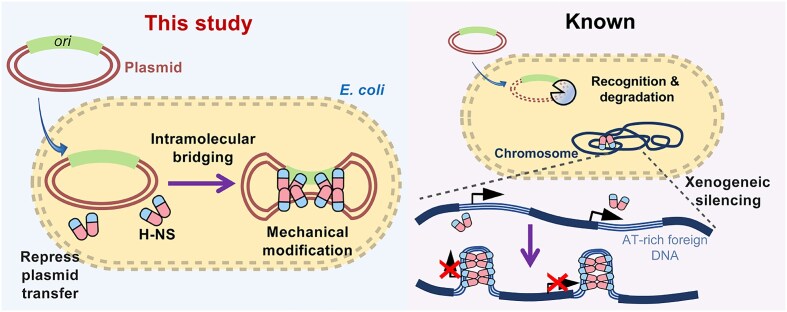

## Introduction

The evolutionary arms race between prokaryotes and invasive mobile genetic elements (MGEs) exerts a powerful evolutionary force that leads to the emergence of a myriad of host-defense systems that protect against invading MGEs [1, 2]. These defense systems include restriction-modification (R-M) [3–7], CRISPR–Cas [8–10], Argonaute [11–13], Gabija [14–19], CBASS [20–23], Shedu [24], Lamassu [25], and Wadjet systems [26–29], which detect invading DNA and consequently initiate defense responses by triggering cell death and/or cleaving detected DNA. Plasmids, which are widely present in prokaryotes, serve as common vectors for horizontal gene transfer [30–33] and are frequently targeted by these host-defense systems [26–29, 34–37]. The four-gene Wadjet system recognizes DNA topology and specifically cleaves closed-circular DNA to protect its host against plasmid transformation [26–29]. Recently, two DNA defense modules, DdmABC and DdmDE, were discovered to work in tandem to eliminate plasmids by triggering cell suicide and degrading DNA, respectively, in the seventh-pandemic *Vibrio cholerae* strain [34–37]. Understanding how bacterial hosts monitor and defend against plasmids is crucial for elucidating the evolutionary and ecological dynamics of plasmid dissemination, which can implement physiology to enable adaptation, such as antibiotic resistance.

Nucleoid-associated proteins (NAPs) play a central role in chromosome organization, DNA replication and repair, as well as transcription regulation in response to environmental cues [38–44]. Similar to many host defense proteins, NAPs can bind double-stranded DNA (dsDNA) [40]. However, unlike known defense systems, which cleave invading nucleic acids or induce bacterial suicide [8, 36, 45], many NAPs function through mechanically bending, bridging, or condensing chromosomal DNA [46, 47]. For example, H-NS, a highly abundant NAP (∼20 000 molecules per cell) [48], preferentially binds to AT-rich dsDNA, a hallmark of horizontally acquired loci [49–55]. The binding of H-NS to specific DNA sequences is followed by its oligomerization along the helix and DNA bridging, leading to the formation of protein–DNA filaments and chromatin remodeling [46, 56–63]. This blocks RNA polymerase from accessing template DNA [64–66]. Consequently, H-NS proteins suppress the transcription of horizontally transferred genes, serving as a xenogeneic silencer (XS) [67–72]. By bridging DNA, H-NS can also direct transposition to maximize evolutionary outcomes for the host cell [73]. Additionally, H-NS and other NAPs are known to bind DNA and alter its mechanical properties [42, 47]. Although the formation of supramolecular assemblies has recently emerged as a common strategy in bacterial immunity [74], it remains unexplored whether the assemblage of NAPs on DNA contributes to host defense before their stable integration into the host cells.

Natural transformation consists of two distinct phases: the uptake of DNA into the cytoplasm and the subsequent processing of the internalized DNA within the cytoplasm [75, 76]. Generally, plasmid DNA is transferred into recipient cells as single-stranded DNA (ssDNA) through conserved membrane-associated DNA transport systems during both natural transformation and conjugation [75, 77]. Our previous work showed that *Escherichia coli* can develop natural competence to take up plasmid DNA on solid agar plates [78, 79]. Unlike other naturally transformable bacteria, *E. coli* takes up DNA in the form of dsDNA under natural conditions [79]. This process is regulated by the stationary-phase regulator RpoS [80], a sigma factor that controls the expression of *ydcS* and *ydcV*, which are believed to encode proteins responsible for the transport of dsDNA [81]. The membrane protein OmpA can compete for dsDNA, thereby blocking plasmid transfer during the natural transformation of *E. coli* [82]. However, the exact mechanism by which internalized plasmid DNA is processed inside *E. coli* cells remains unknown.

In this study, we have shown that H-NS plays a crucial role in the processing of internalized plasmid DNA during natural transformation in *E. coli*. We propose that the mechanical modification of plasmid DNA by H-NS may represent a novel mechanism for host defense. This study expands our understanding of the functional diversity of NAPs and highlights the broader implications of NAP-mediated DNA modification for bacterial immunity, offering new insights into how bacteria may defend themselves against MGEs.

## Materials and methods

### Bacterial strains, plasmids, oligonucleotides, growth conditions, and media

Bacterial strains and plasmids used in this study are listed in Supplementary Tables S1 and S2, respectively. Oligonucleotides are listed in Supplementary Table S3. Recombinant strains were constructed using the λ-derived Red recombination system [83]. Recombinant plasmids were constructed using a One-Step PCR Cloning kit. Details of the construction of strains and plasmids are described in the Supplementary methods. *Escherichia coli* was grown at 30°C or 37°C in LB (Luria–Bertani) or M9 minimal medium [84] supplemented with 1% (wt/vol) glucose as the carbon source, with shaking at 200 rpm, or on LB agar plates containing 2% (wt/vol) or 5% (wt/vol) agar. When required, antibiotics and supplements were added to the media, including ampicillin (100 μg/mL), kanamycin (50 μg/mL), chloramphenicol (25 μg/mL), and arabinose (20 mM).

### Natural transformation of *E. coli*

Natural transformation of *E. coli* was performed according to a documented method [84] with slight modifications. Briefly, overnight-grown *E. coli* cells were inoculated into fresh M9 medium supplemented with 1% (wt/vol) glucose and incubated at 30°C with shaking for 20 h, reaching an optical density of 600 nm (OD_600_) of 2.0–2.5. Then, 1 mL of cell culture was sedimented and the precipitated cells were resuspended in 100 μL of M9 medium. Plasmid was added to the cell suspension solution to achieve a final concentration of 40 μg/mL. Cell mixture was spread onto an LB agar (5% wt/vol) plate (BD Difco) supplemented with 150 μg/mL ampicillin and incubated at 30°C for 1–2 days. Colony-forming units (CFUs) of the transformants were counted. Transformation frequency (TF) was defined as the ratio of the number of transformants divided by the number of counts of viable cells.

### Electrophoretic mobility shift assay

Electrophoretic mobility shift assay (EMSA) was performed as documented [63]. Probes (1 ng/μL) were mixed with purified H-NS protein at different concentrations in the presence of binding buffer (10 mM Tris–HCl, pH 7.9, 125 mM KCl, 10 mM MgCl_2_, 0.1 mM DTT, 2.5% glycerol). The mixture was incubated for 30 min at room temperature and then separated by electrophoresis at 100 V for 45 min in 5% native polyacrylamide gels in 0.5 × TBE solution. Gels were stained with SYBR Gold staining using an orbital shaker at 50 rpm for ∼20 min and visualized using an iBright 1500 scanner (Invitrogen). The motif and motif* probes were generated by polymerase chain reaction (PCR) amplification with the primer pair P_15_–P_16_ using pGLO-motif and pGLO-motif* as templates, respectively (Supplementary Table S3). The dsDNA probe containing H-NS binding site (HBS) was generated by annealing with the primer pair P_21_–P_22_ (Supplementary Table S3).

### Bacterial conjugation assay

Bacterial conjugation was performed as described previously [85], using *E. coli* WM3064 containing pHG101-motif, pHG101-motif*, or pMD20-*mob* as donor strains, and *E. coli* BW25113 and the Δ*hns* mutant as recipient strains. Donor WM3064 strains are auxotrophic for diaminopimelic acid (DAP) and grow in LB supplemented with kanamycin (10 μg/mL) and DAP (57 μg/mL). When separately grown to an OD_600_ of 0.6, recipient (750 μL) and donor (1500 μL) cell cultures were sedimented and resuspended in 100 μL LB, then plated on the LB agar plate with 57 μg/mL DAP and incubated at 30°C for 6 h. Then, cells on the plate were resuspended with 1 mL LB, followed by serial dilution and spreading on LB agar plates supplemented with or without 10 μg/mL kanamycin. After incubation, the CFUs of recipients (on plates without kanamycin) and transconjugants (on plates with kanamycin) were counted. Conjugation frequency was defined as the number of transconjugants divided by the number of recipient cells.

### DNA-bridging assay

DNA-bridging assay was performed by using the method described previously [86], with modifications as follows. The DNA probes were prepared by PCR amplification using 5′-Cy5 or 5′-biotin-labeled primers as listed in Supplementary Table S3. For the DNA-bridging assay, 200 μL Streptavidin Magbeads were washed twice with 500 μL PBS and then resuspended in 500 μL Coupling Buffer (CB: 20 mM Tris–HCl, pH 7.4, 1 mM ethylenediaminetetraacetic acid, 500 mM NaCl). Next, 2.5 μg of biotin-labeled fragment I was added into the suspension and incubated with the beads for 30 min at room temperature with gentle rotation. The beads were washed twice with 500 μL Incubation Buffer (IB: 20 mM Tris–HCl, pH 7.4, 150 mM NaCl, 1 mM dithiothreitol, 5% glycerol (vol/vol), 0.05% Tween 20) and resuspended in IB supplemented with 2.5 μg Cy5-labeled fragment II to a final volume of 300 μL, and then divided into 50 μL aliquots. Two-fold serially diluted protein samples were added into each of the 50 μL aliquots to a final volume of 60 μL. After a 30 min incubation at room temperature with gentle rotation, the mixture was placed on a magnetic stand for separation. The supernatant was transferred into a new tube and marked as sample A. The beads were washed and incubated in 60 μL elution buffer [EB: IB supplemented with 0.1% sodium dodecyl sulfate (SDS) and 20 μg/mL biotin] and boiled for 10 min. The eluted sample was transferred into a new tube and marked as sample B. The Cy5 fluorescence signals of sample A and sample B were detected by an iBright 1500 scanner (Invitrogen) and quantified by using ImageJ. The Cy5 fluorescence signal of sample A from the reaction without adding protein was defined as 100% input signal. Sample A was also subjected to EMSA to check the binding of free fragment II and H-NS protein.

### Plasmid DNA-bridging assay

The plasmid DNA-bridging assay was performed by using a previously described method [87], with modifications as follows. One hundred fifty microliters of Streptavidin Magbeads were washed twice with 500 μL PBS and then resuspended in 500 μL CB. The suspension was incubated with 2 μg biotin-labeled DNA for 30 min at room temperature. Beads were collected by a magnetic stand, washed three times with IB, and resuspended in IB supplemented with 2 μg plasmids to a final volume of 200 μL. The bead suspension was then divided into 50 μL aliquots. Two-fold serially diluted protein samples were added into each of the 50 μL aliquots to a final volume of 60 μL. After a 30 min incubation at room temperature, the beads were collected by magnet and washed twice with IB. H-NS was removed from the reaction by adding 10 μg/μL proteinase K and 0.5% SDS for 30 min at 65°C. Then the beads were collected by magnet, and the supernatant was resolved by 1% TAE agarose gel electrophoresis. The amounts of bridged plasmid were quantified by using ImageJ.

### Transmission electron microscopy

Transmission electron microscopy (TEM) was performed by using the method described previously [88], with modifications as follows. H-NS protein (0.5 μg/μL) and plasmid (1 μg/μL) were co-incubated at room temperature for 20 min. The sample was deposited on a carbon-coated copper grid (GilderGrids) and left to evaporate at room temperature. Then the sample was stained with 2% sodium phosphotungstic acid (pH 7.0) for 1 min and dehydrated by air. Finally, the sample was photographed using a Hitachi HT7700 EXALENS transmission electron microscope operated at 120 kV.

### Statistical analysis

All statistical details of experiments can be found in the figure legends. All experiments were repeated with at least three biological replicates. All statistics were performed by using GraphPad Prism. To determine statistical significance, Student’s *t*-test was performed for pairwise comparisons, and one-way ANOVA with Dunnett’s multiple comparisons test was utilized for comparisons between multiple groups. Values are reported as mean ± SD. For TF test panels, the data are shown as the geometric mean ± geometric SD.

## Results

### H-NS and its family proteins reduce plasmid transfer during natural transformation of *E. coli*

**Figure 1.**
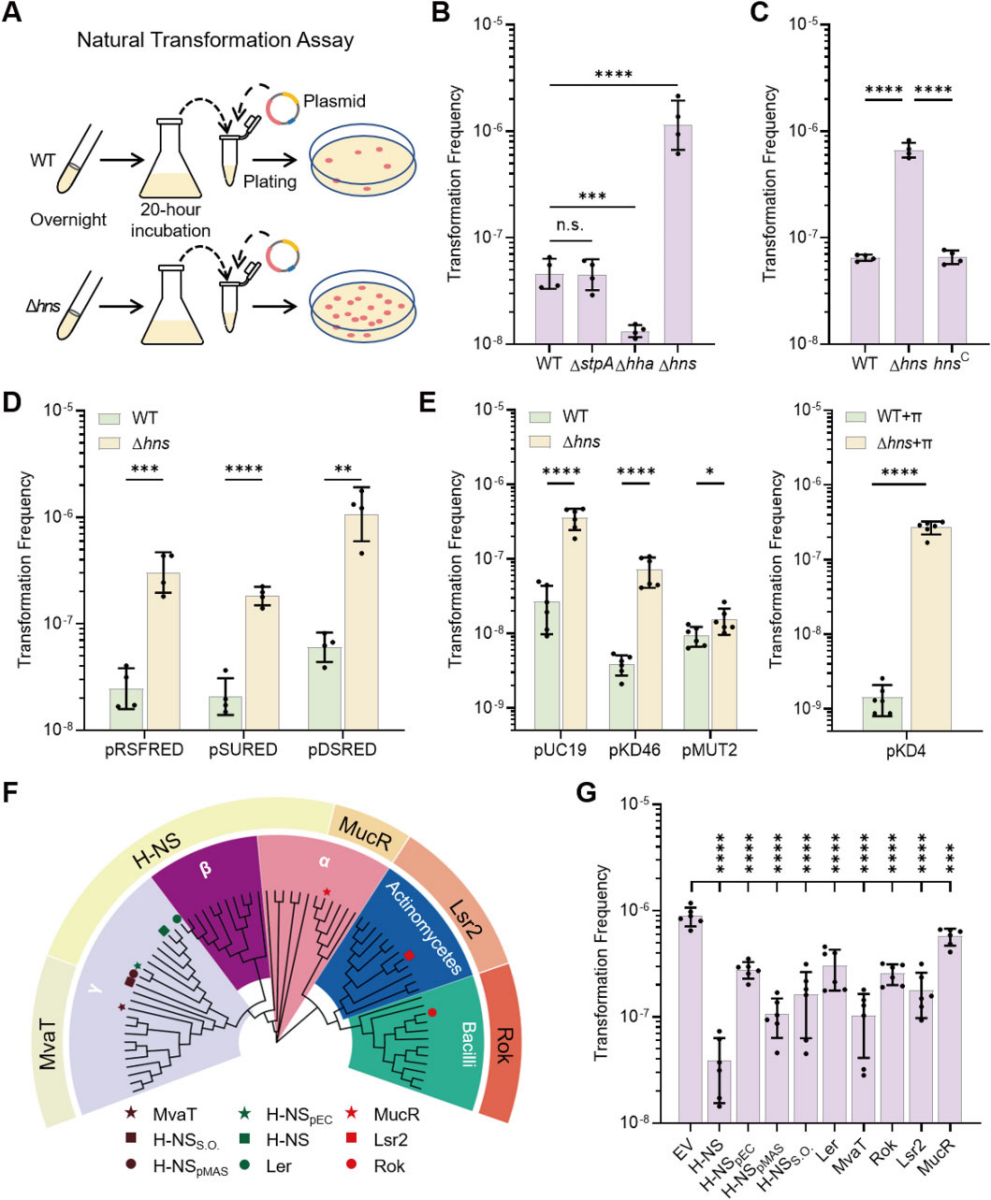
H-NS suppresses plasmid transfer during natural transformation. (**A**) Schematic diagram of plasmid transfer assay. Natural transformation was performed as follows. Overnight-grown *E. coli* cells were inoculated into fresh M9 medium and incubated at 30°C with shaking for 20 h. After incubation, the cells from 1 mL of culture were harvested by centrifugation and resuspended in 100 μL of M9 medium. Plasmid DNA was then added to the cell suspension, and the mixture was spread on LB plates containing 5% (wt/vol) agar, supplemented with the appropriate antibiotics. The level of plasmid transfer of strains is evaluated by TF. The detection limit of natural transformation is ∼10^−10^. (**B**) TFs of Δ*stpA*, Δ*hha*, and Δ*hns* mutants were compared to the WT strain BW25113 (*n* = 4). (**C**) Complementation analysis of H-NS on plasmid transfer. TF was assayed in the Δ*hns* mutant, the corresponding complementary strain (*hns*^C^), and WT, with pDSRED as the donor DNA (*n* = 4). (**D**) TFs of the Δ*hns* mutant and WT were evaluated with plasmids of different ColE1 replicons, including pDSRED (pUC19-based, pMB1 replicon), pRSFRED (pRSF replicon), and pSURED (p15A replicon) (*n* = 4). (**E**) TFs of the Δ*hns* and WT strains were evaluated with plasmids pUC19 (pMB1 replicon, Class B θ replicon), pKD46 (pSC101 replicon, Class A θ replicon), pMUT2 (ColE2 replicon, Class C θ replicon), and pKD4 (R6K replicon, Class A θ replicon) (*n* = 6). Plasmid pKD4 was transformed to Δ*hns* and WT expressing π replication initiator protein. (**F**) Phylogenetic tree of the xenogeneic silencers from different hosts. Color palettes indicate host classifications, and the outer rings indicate xenogeneic silencer classifications. MvaT is derived from γ-Proteobacteria species *Pseudomonas aeruginosa*. H-NS_S.O._ is derived from γ-Proteobacteria species *Shewanella oneidensis*. H-NS_pMAS_ and H-NS_pEC_ are from MGEs, which are encoded by plasmids from γ-Proteobacteria species *Escherichia coli*. H-NS is derived from γ-Proteobacteria species *Escherichia coli*. Ler is derived from γ-Proteobacteria species *Escherichia coli* O157:H7 str. Sakai. MucR is derived from α-Proteobacteria species *Sinorhizobium fredii*. Lsr2 is derived from Actinomycetes species *Mycobacterium tuberculosis*. Rok is derived from Bacilli species *Bacillus subtilis*. Functions of the NAPs in the key are examined through natural transformation, as shown in panel (G). (**G**) Heterologous expression of xenogeneic silencers inhibits plasmid transfer. TF levels were measured in the Δ*hns* with plasmids expressing different xenogeneic silencers, including pSU-P_BAD_-*hns* (H-NS), pSU-P_BAD_-*hns*_pEC_ (H-NS_pEC_), pSU-P_BAD_-*hns*_pMAS_ (H-NS_pMAS_), pSU-P_BAD_-*ler* (Ler), pSU-P_BAD_-*hns*_S.O._ (H-NS_S.O._), pSU-P_BAD_-*mvaT* (MvaT), pSU-P_BAD_-*rok* (Rok), pSU-P_BAD_-*lsr2* (Lsr2), and pSU-P_BAD_-*mucR* (MucR), with the empty vector pSU19 (EV) as the control. These strains were grown at 30°C for 20 h in M9 minimal medium supplemented with 20 mM arabinose before transformation (*n* = 6). pEC14_35 and pMAS2027 are two plasmids expressing H-NS family proteins H-NS_pEC_ and H-NS_pMAS_ with GenBank accession numbers AFC60903.1 and ACV89896.1, respectively. Statistical significance was assessed using a two-tailed Student’s *t*-test (* *P* ≤ .05, ** *P* ≤ .01, *** *P* ≤ .005, **** *P* ≤ .001). Data are shown as the geometric mean ± geometric SD.

H-NS is an abundant protein that is extensively associated with bacterial chromosomal DNA [40]. *Escherichia coli* naturally takes up plasmids in the form of dsDNA [75, 78, 79]. To assess the role of H-NS in plasmid transfer during natural transformation in *E. coli*, we first evaluated the impact of *hns* gene deletion (Fig. 1A). Deletion of the *hns* gene resulted in a ∼10-fold increase in TF with pDSRED, a pMB1 (ColE1-type) derivative plasmid, as the donor DNA (Fig. 1B). Inactivating *hha*, the accessory protein of H-NS, significantly (albeit only slightly) reduced TF (Fig. 1B). In contrast, inactivating *stpA*, the homologue of *hns*, did not affect natural transformation (Fig. 1B). The genomic *hns* complementation (*hns*^C^) in the Δ*hns* mutant reduced TF to the wild-type (WT) level (Fig. 1C). The increased TF resulting from *hns* inactivation was consistently observed across plasmids with replicons from different classes, including three other ColE1 plasmids (pUC19, pSURED, and pRSFRED), a pSC101 plasmid (pKD46), an R6K plasmid (pKD4), and a ColE2 plasmid (pMUT2) (Fig. 1D and E and Supplementary Fig. S1A). Notably, pKD46 and pKD4, which possess replicons with high AT-content, exhibited relatively low TFs in the WT strain (Fig. 1D and E and Supplementary Fig. S2), suggesting that the replicon AT-content is a critical factor in H-NS-mediated repression of plasmid transfer.

H-NS family proteins are widely distributed in MGEs and α-, β-, and γ-Proteobacteria (Fig. 1F). We observed that H-NS, from the plasmids pMAS2027 (H-NS_pMAS_) and pEC14_35 (H-NS_pEC_), exhibited significant suppression of plasmid transfer in the Δ*hn*s mutant, reducing TFs by 7.4- and 2.2-fold, respectively (Fig. 1G). H-NS family proteins from *S. oneidensis* (H-NS_S.O._) also suppressed plasmid transfer in the Δ*hn*s mutant, reducing TFs by 4.4-fold (Fig. 1G). Considering that H-NS has been known to function as an XS, we examined functions of non-homologous XSs MvaT, Lsr2, Rok, and MucR from γ-Proteobacteria, Actinomycetes, Bacilli, and α-Proteobacteria, respectively, in complementing the defense function of H-NS. MvaT, Rok, and Lsr2 displayed strong suppression effects, reducing TFs by 7.7-, 2.5-, and 4.0-fold, respectively. Whereas, MucR had a slight but significant effect on limiting plasmid transfer in the Δ*hns* mutant (Fig. 1G). Furthermore, Ler, an antagonist of H-NS from *E. coli* O157:H7 str. Sakai, also suppressed plasmid transfer in the Δ*hn*s mutant, reducing TFs by 1.9-fold (Fig. 1G). These findings reveal a broad capacity for XSs from distantly related species and MGEs to suppress plasmid transfer.

The observed reduction in TFs for multiple NAPs may be attributed to the fact that alterations in NAPs induce changes in chromosomal architecture, which could in turn affect interactions between the chromosome and plasmid. These NAP-mediated chromosomal structural changes might potentially influence transformation [89]. To test this possibility, we examined the effect of MatP, which affects chromosome segregation but is not classified as a NAP protein [90, 91], on natural transformation. The results showed that inactivation of *matP* did not significantly impact plasmid transformation (Supplementary Fig. S3), indicating that simply altering chromosomal structure does not play a role in reducing plasmid transfer.

### H-NS limits plasmid transfer by directly interacting with transforming dsDNA

**Figure 2.**
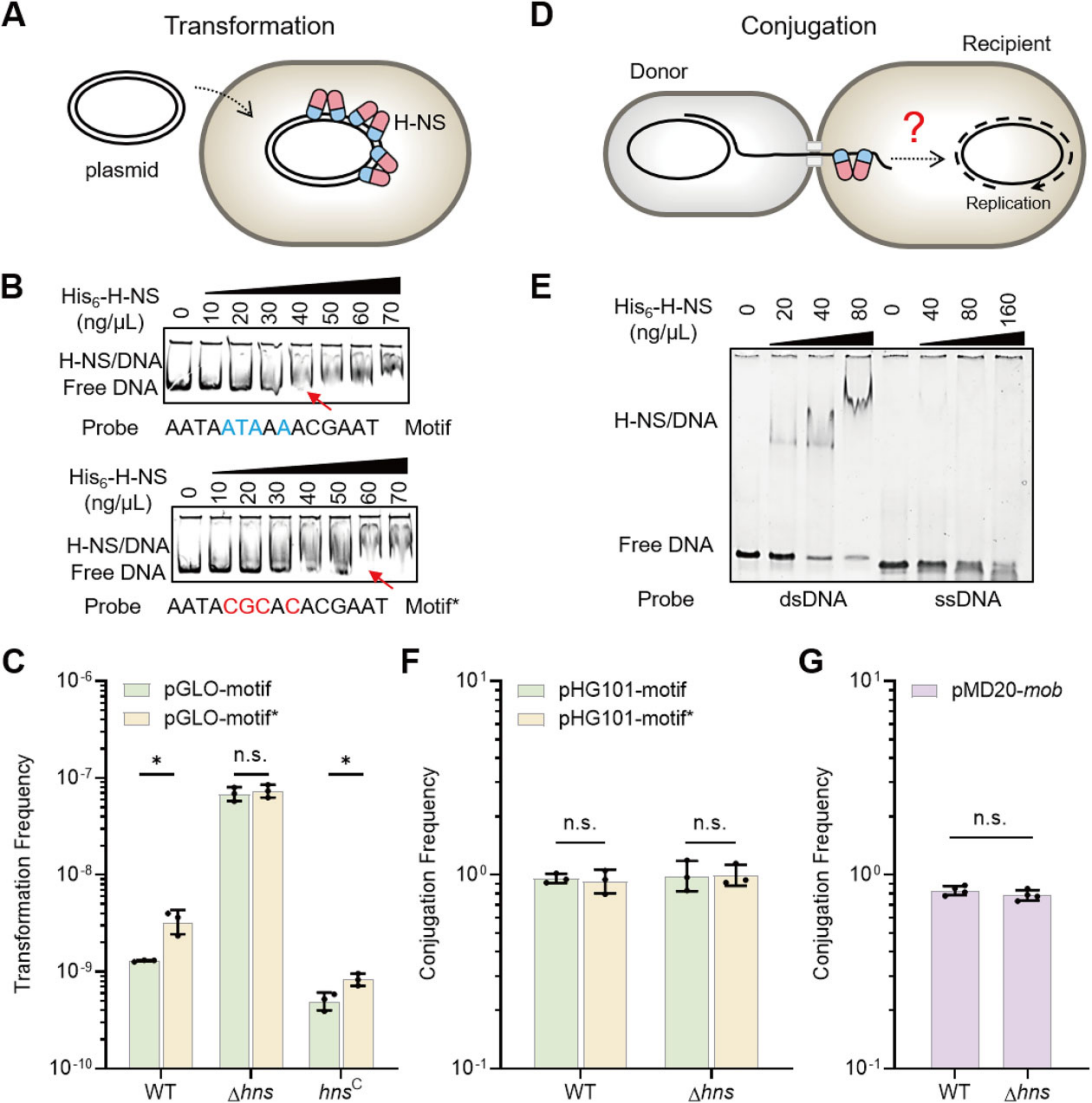
H-NS reduces plasmid transfer in the form of dsDNA during natural transformation but not ssDNA during conjugation. (**A**) Schematic representation of H-NS interaction with transferring dsDNA during natural transformation. (**B**) EMSA with probes containing the intact motif or mutated motif*. The sample was separated with 5% native polyacrylamide gels. The motif and motif* have intact and mutated HBS, respectively. Probe concentration: 1 ng/μL. The numbers above the lanes represent the His_6_-H-NS protein concentrations used. The red arrows mark the minimum protein concentration required for detectable binding to free DNA. (**C**) TFs were measured in the WT, Δ*hns*, and the complemented strain (*hns*^C^) using plasmids pGLO-motif and pGLO-motif* (*n* = 3). (**D**) Schematic representation of putative interaction between H-NS and transferring ssDNA during conjugation. (**E**) EMSA for detecting interactions between His_6_-H-NS protein and ssDNA/dsDNA probes containing HBS. Samples were separated with 5% native polyacrylamide gels. His_6_-H-NS protein concentrations: 0, 20, 40, and 80 ng/μL for dsDNA, and 0, 40, 80, and 160 ng/μL for ssDNA. Probe concentration: 1 ng/μL. (**F**) Conjugation of the mobilizable plasmid pHG101-motif or pHG101-motif* from the donor strain WM3064 to the recipient strain Δ*hns* or WT (*n* = 3). (**G**) Conjugation of pMD20-*mob* (pMB1 derivative mobilizable plasmid) from the donor strain WM3064 to the recipient strain Δ*hns* or WT (*n* = 4). Statistical significance was assessed using a two-tailed Student’s *t*-test (* *P* ≤ .05, n.s. *P* > .05). Data are shown as the geometric mean ± geometric SD.

H-NS has been shown to directly interact with and compact dsDNA *in vitro* [92], and its transcriptional suppression effect is associated with the presence of HBS [51]. To investigate whether H-NS inhibits plasmid transfer by directly interacting with transferring dsDNA during natural transformation (Fig. 2A), we generated a plasmid pGLO-motif containing an intact HBS downstream of the replicon and a plasmid pGLO-motif* containing a mutated HBS. EMSA experiment showed that mutation of HBS reduced the affinity between H-NS and the probe DNA (Fig. 2B). In the WT, the TF with pGLO-motif was 1.29 × 10^−9^, significantly lower than that with pGLO-motif* (3.33 × 10^−9^), indicating that HBS mutation promotes plasmid transfer (Fig. 2C). By contrast, in the Δ*hns* mutant, no significant difference in TFs was detected between the use of pGLO-motif and pGLO-motif*, indicating that the affinity between H-NS and HBS on the plasmid is required for limiting plasmid transfer (Fig. 2C). Complementation of the Δ*hns* mutant with a functional *hns* gene restored the inhibitory effect of H-NS on plasmid transfer. The suppression effect of H-NS on transfer of pGLO-motif was apparently stronger than that of pGLO-motif* in the complemented strain (Fig. 2C), confirming that H-NS–DNA interactions play an important role in inhibiting plasmid transfer.

An additional, non-mutually exclusive mechanism by which H-NS may suppress plasmid transfer is through the transcriptional regulation of host genes involved in transformation. H-NS has been shown to reduce levels of RpoS, a competence regulator in *E. coli* [80]. However, inactivating *hns* significantly reduced TFs in the Δ*rpoS* strain (Supplementary Fig. S4), indicating that H-NS inhibits plasmid transfer via an RpoS-independent pathway. Notably, deletion of *ydcS* and *ydcV*, which likely play roles in transporting plasmid DNA [81], significantly decreased TFs in the WT, whereas their inactivation had little impact on TFs in the Δ*hns* mutant (Supplementary Fig. S5). This observation suggests that, in the absence of H-NS, expression of other competing proteins may mitigate the role of YdcS and YdcV. Interestingly, disruption of *ompA*, which encodes a competing protein [82], led to a significant increase in TFs in both WT and Δ*hns* strains (Supplementary Fig. S5), implying that OmpA is not involved in H-NS-mediated repression of plasmid transfer. We examined the involvement of several H-NS-regulated genes encoding membrane-associated proteins (*ydfJ*, *ynaI*, *cusC*, *ybgQ*, *htrE*, *yehB*, and *ompN*) in plasmid transfer during natural transformation. However, none of these genes appeared to influence transformation under our experimental conditions (Supplementary Fig. S6).

To determine whether H-NS represses plasmid transfer in the form of ssDNA during conjugation (Fig. 2D), we generated two mobilizable plasmids, pHG101-motif with an intact HBS and pHG101-motif* with a mutated HBS. Previous studies have shown that H-NS preferentially binds dsDNA over ssDNA [93]. Our EMSA experiment confirmed that H-NS bound the dsDNA probe with high affinity but hardly bound the ssDNA probe (Fig. 2E), despite both probes containing HBS. Transfer rates of pHG101-motif and pHG101-motif* were similar in both WT and the Δ*hns* mutant strains (Fig. 2F), indicating that H-NS does not affect plasmid transfer during conjugation. To validate this, we generated another mobilizable plasmid, pMD20-*mob*, with essential conjugation elements (*oriT* and the gene encoding DNA relaxase) inserted into a pMB1 derivative plasmid. Conjugation rates with pMD20-*mob* were still similar in the WT and Δ*hns* mutant (Fig. 2G). These results clearly show that H-NS does not suppress plasmid transfer in the form of ssDNA.

### Both DNA binding and polymerization of H-NS are essential for the limitation of plasmid transfer

**Figure 3.**
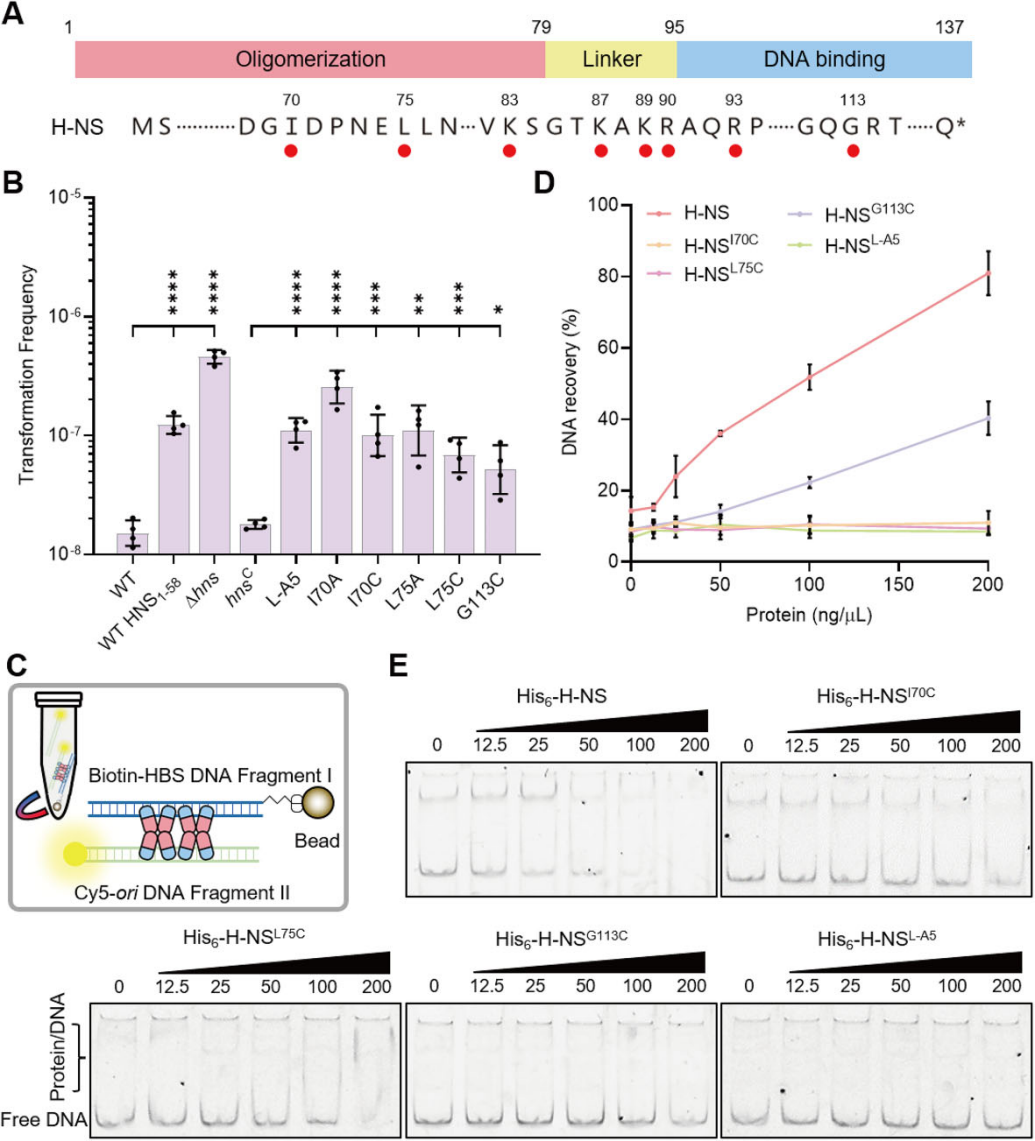
The DNA bridging ability of H-NS is necessary to repress plasmid transfer. (**A**) Schematic diagram of H-NS domain organization and mutated positions. H-NS consists of an oligomerized N-terminal domain (NTD) and a DNA-binding C-terminal domain (CTD), connected by a flexible linker region. The substituted residues are marked with circles. (**B**) TFs of WT, WT H-NS_1–58_, Δ*hns*, L-A5, I70A, I70C, L75A, L75C, and G113C with pDSRED as donor plasmid. WT H-NS_1–58_ was generated by integrating a truncated *hns* gene encoding the N-terminal fragment (residues 1–58). The L-A5, I70A, I70C, L75A, L75C, and G113C were compared with the complemented strain *hns*^C^, while the Δ*hns* and WT H-NS_1–58_ were compared with the WT, with pDSRED as the donor DNA (*n* = 4). Statistical significance of TF was assessed using a one-way ANOVA with Dunnett’s multiple comparisons test against the *hns*^C^ (* *P* ≤ .05, ** *P* ≤ .01, *** *P* ≤ .005, **** *P* ≤ .001). Data are shown as the geometric mean ± geometric SD. (**C**) Schematic illustration of DNA-bridging assay. H-NS bridges the biotin-labeled probe anchored to streptavidin magnetic bead and the Cy5-labeled probe in solution. The fragment I containing the HBS is labeled with biotin, and the fragment II containing the replicon of the pUC19 is labeled with Cy5. (**D**) DNA recovery (as a percentage of all Cy5-labeled DNA) as a function of concentrations of H-NS, H-NS^I70C^, H-NS^L75C^, H-NS^G113C^, or H-NS^L-A5^ was measured by the DNA bridging assay (*n* = 3). Fragments I and II were used for DNA-bridging assay. Data are shown as the mean ± SD. (**E**) EMSA of DNA-bridge assay supernatant. EMSA shows the affinity of fragment II with H-NS or its variants (His_6_-H-NS^I70A^, His_6_-H-NS^I70C^, His_6_-H-NS^L75A^, His_6_-H-NS^L75C^, and His_6_-H-NS^L-A5^). Sample A of the DNA-bridging assay was separated with 5% native polyacrylamide gels. Protein concentrations: 0, 12.5, 25, 50, 100, and 200 ng/μL. The Cy5-labeled fragment II concentration: 5 ng/μL.

H-NS comprises two functional domains: the N-terminal oligomerization domain (residues 1–79) and the C-terminal DNA-binding domain (residues 96–137), connected by a flexible linker (Fig. 3A) [53, 61]. The G113 residue in the C-terminal domain is required for the DNA-binding activity of H-NS [63, 94]. Our results showed that the H-NS^G113C^ variant had a reduced affinity for a DNA probe containing the HBS *in vitro* (Supplementary Fig. S7). To assess the impact of the DNA-binding activity of H-NS on plasmid transfer, we constructed a strain expressing the H-NS^G113C^ variant from the native chromosomal locus by allelic replacement. Compared to the strain expressing WT H-NS (*hns*^C^), the TF of the mutant expressing H-NS^G113C^ was increased by 2.1-fold (Fig. 3B). This finding indicates that reducing the DNA-binding activity of H-NS alleviates its inhibition of plasmid transfer.

Residues I70 and L75, located within the N-terminal oligomerization domain of H-NS, are important for the formation of rigid and extended H-NS complexes along DNA [63]. To assess whether the stiffened filament hinders plasmid transfer, the I70 and L75 residues were substituted with alanine or cysteine, resulting in strains expressing the H-NS^I70A^, H-NS^I70C^, H-NS^L75A^, and H-NS^L75C^ variants from the native chromosomal locus by allelic replacement. TFs of all of these strains were 3.0- to 13.8-fold higher than that of *hns*^C^ (Fig. 3B), indicating that the formation of the stiffened H-NS:DNA filament is crucial for suppressing plasmid transfer.

The linker region, which connects the N-terminal and C-terminal domains of H-NS, has recently been shown to facilitate both H-NS binding to and polymerizing on DNA through its positively charged residues K83, K87, K89, R90, and R93 [61]. To investigate the role of this linker region in H-NS-mediated suppression of plasmid transfer, these residues were substituted by alanine, yielding an *hns* mutant (L-A5) that expressed the H-NS^K83A, K87A, K89A, R90A, R93A^ variant (H-NS^L-A5^) from the native chromosomal locus by allelic replacement. The TF of L-A5 was 1.13 × 10^−7^, 5.3-fold higher than that of *hns*^C^ (Fig. 3B), revealing that the linker region is essential for H-NS function in repressing plasmid transfer. These findings further support that H-NS inhibits plasmid transfer by forming a higher-order DNA-H-NS complex.

### H-NS mediates bridging between the plasmid replicon and an H-NS-binding DNA fragment

Given that H-NS preferentially binds to the AT-rich region, a characteristic feature of bacterial replication origins [95], we hypothesized that the interaction between H-NS and the replicon might play a significant role in suppressing plasmid transfer. To test this hypothesis, we fragmented the plasmid pUC19 into six segments and assessed the binding affinity of H-NS to each fragment by using EMSA. H-NS preferentially bound to the fragments overlapping the replicon region, which exhibits relatively low GC content (Supplementary Fig. S8). Notably, H-NS proteins have been shown to bridge distant DNA segments together [62, 65], thus may have bridged the replicon with other regions on the plasmid during this process. To check this hypothesis, we performed an intermolecular DNA-bridging assay. In this assay, the biotin-labeled DNA probe containing the HBS fragment (fragment I) was associated with the streptavidin magnetic beads, while the Cy5-labeled DNA probe containing the pMB1 replicon (fragment II) was bridged to bead-associated fragment I by H-NS (Fig. 3C). The sequences of probes are provided in the Supplementary methods. The formation of DNA bridges between fragments I and II by H-NS or its variants was analyzed by measuring the Cy5 fluorescence signal (Fig. 3D and Supplementary Fig. S9). Our results confirmed that H-NS facilitated bridging between the DNA fragment containing the HBS and the replicon (Fig. 3D).

We further evaluated the bridging activity of H-NS variants, which are defective in repressing plasmid transfer. When the concentration of the WT H-NS exceeded 100 ng/μL, the band of free Cy5-labeled probe (fragment II) became weak (Fig. 3E). In contrast, the H-NS^G113C^ variant exhibited severe defects in bridging the HBS and the replicon (Fig. 3D) as well as in binding to the fragment II, as no obvious reduction in the probe band was observed even at 200 ng/μL H-NS (Fig. 3E). Similarly, the H-NS^L-A5^ variant lost its ability to bridge the HBS and the replicon (Fig. 3D) and to bind the fragment II (Fig. 3E). Additionally, the H-NS^I70C^ and H-NS^L75C^ variants abolished the ability to bridge fragments I and II (Fig. 3D) while retaining a slight DNA binding capacity to fragment II (Fig. 3E). Our results demonstrate that H-NS variants defective in repressing plasmid transfer are impaired in DNA bridging.

### H-NS suppresses plasmid transfer by forming intramolecular DNA bridges within the plasmid

A previous study demonstrated that the H-NS_1–58_ variant disrupts the DNA-bridging of WT H-NS, acting as an H-NS bridging “inhibitor” [62], which is consistent with the result of our DNA-bridging assay (Supplementary Fig. S10). To investigate the impact of H-NS-mediated DNA bridging ability on plasmid transformation, the H-NS_1–58_ variant was ectopically expressed in the WT BW25113 strain. The TF of the strain expressing the H-NS_1–58_ variant (WT H-NS_1–58_) was significantly higher than that of the strain not expressing it (Fig. 3B). Together with the observed effects of H-NS^I70C^, H-NS^L75C^, H-NS^G113C^, and H-NS^L-A5^ on plasmid transfer shown in Fig. 3B, our results indicate that H-NS-mediated intramolecular DNA bridging is likely required for repression of plasmid transfer during natural transformation.

**Figure 4.**
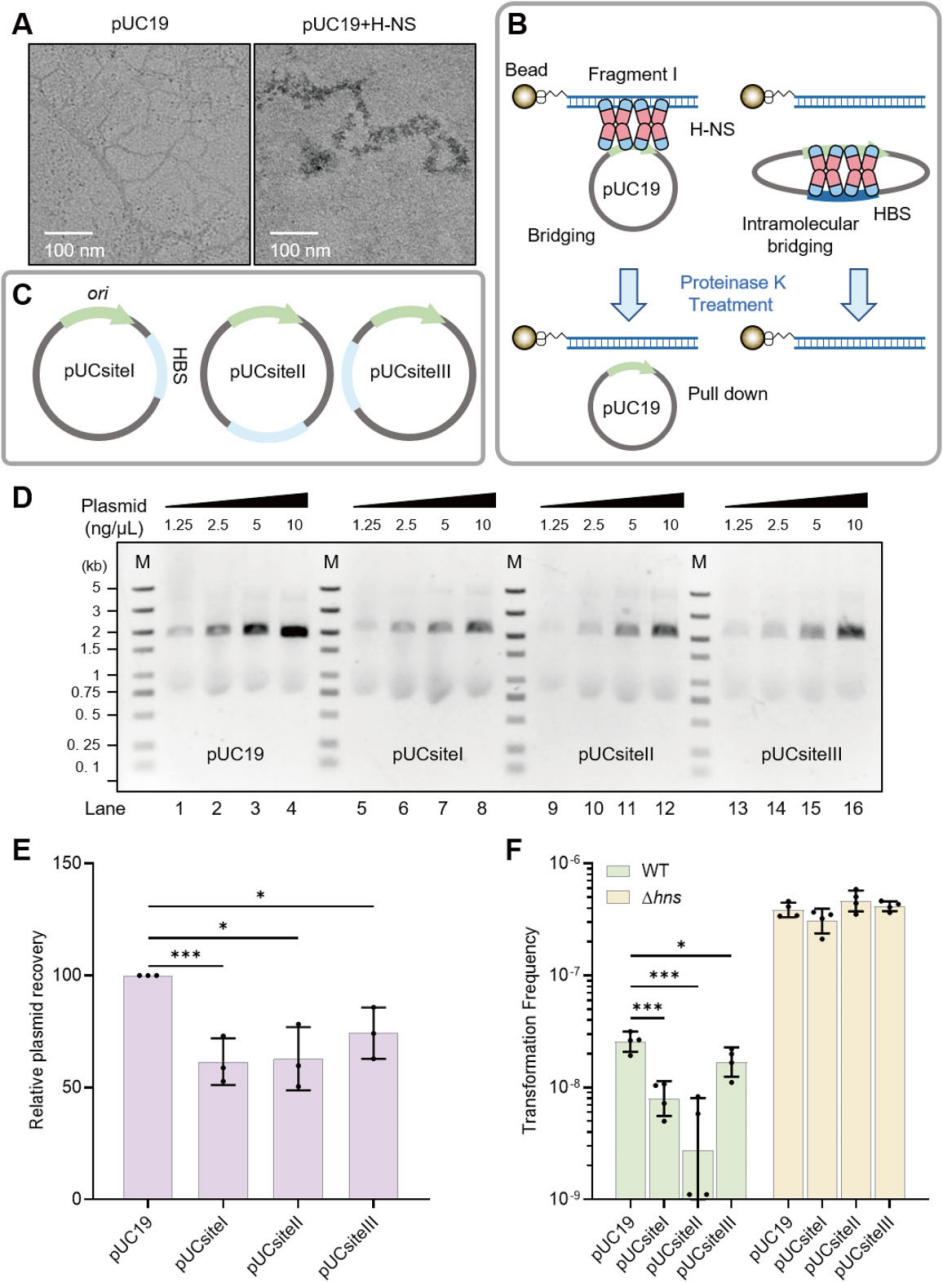
Positional effect of HBS on plasmid transfer. (**A**) TEM analysis of the structures of naked pUC19 (left) and H-NS-bound pUC19 (right). (**B**) Schematic diagram of plasmid DNA-bridging assay. H-NS bridges the plasmid and biotin-labeled probe anchored to streptavidin magnetic beads in solution. The formation of intramolecular-bridging plasmid mediated by H-NS precludes association with the biotin-labeled probe. Proteinase K is used to remove H-NS and release the bridged plasmid. (**C**) HBS is placed downstream (pUCsiteI), opposite (pUCsiteII), or upstream (pUCsiteIII) of the plasmid replicon. (**D**) Optimization of fragment I/plasmid ratio for the plasmid DNA-bridging assay. A plasmid DNA-bridging assay was performed using 200 ng/μL H-NS protein and 5 ng/μL fragment I. Significant differences in intramolecular bridging between plasmids were observed at fragment I/plasmid ratios of 1:1 and 1:2. The fragment I/plasmid ratios were 4:1 for lanes 1, 5, 9, and 13; 2:1 for lanes 2, 6, 10, and 14; 1:1 for lanes 3, 7, 11, and 15; and 1:2 for lanes 4, 8, 12, and 16. Bridged plasmids were separated by 1% TAE agarose gel electrophoresis. (**E**) The ability of H-NS to bridge plasmids (10 ng/μL) and probes (5 ng/μL) was quantified for pUC19, pUCsiteI, pUCsiteII, and pUCsiteIII (*n* = 3). The amount of plasmids bridged by H-NS was quantified using ImageJ, with the bridged pUC19 plasmid set to 100%. Data are shown as the mean ± SD. (**F**) TFs were measured for WT and Δ*hns* with plasmids pUC19, pUCsiteI, pUCsiteII, and pUCsiteIII (*n* = 4). Data are shown as the geometric mean ± geometric SD. Statistical significance was assessed using a two-tailed Student’s *t*-test (* *P* ≤ .05, ** *P* ≤ .01, *** *P* ≤ .005, **** *P* ≤ .001, n.s. *P* > .05).

Next, we used TEM to examine the effect of H-NS on plasmid structure. In the absence of H-NS, the plasmid displayed a relaxed circular shape. In contrast, the plasmid that had been co-incubated with H-NS was visualized as thick filaments, adopting a more compacted form (Fig. 4A). These observations are consistent with previous atomic force microscopy (AFM) analysis [92]. In contrast, for linear DNA containing known HBSs, although thicker filaments were observed in the presence of H-NS compared to controls lacking H-NS, no compacted regions were detected (Supplementary Fig. S11).

As shown in Fig. 3, H-NS proteins bridge linear DNA fragments containing HBS and the pMB1 replicon. To further investigate whether and how the HBS and the pMB1 replicon form bridges within the circular plasmid DNA, we designed a plasmid DNA-bridging assay. In the presence of H-NS, a DNA bridge would form between the pMB1 replicon and the linear fragment I anchored on beads, enabling bead-based plasmid recovery. On the other hand, if DNA bridges form between the pMB1 replicon and the HBS within the plasmid, the replicon would be concealed, preventing its interaction with fragment I, thereby reducing the fraction of plasmid being recovered by the beads (Fig. 4B).

To test this, we generated three pUC19-derived plasmids by positioning HBS adjacent to the pMB1 replicon either downstream (pUCsiteI), upstream (pUCsiteIII), or opposite to it (pUCsiteII) (Fig. 4C). We observed that H-NS bridged fragment I with all four plasmids in their supercoiled forms without significant differences, indicating a broad impact of H-NS on the bridging of supercoiled DNA (Fig. 4D). However, H-NS differentially bridged fragment I with the four plasmids in the relaxed form. The relaxed form of pUC19, which lacks the HBS, was bridged with fragment I by H-NS and was recovered from the fragment I-H-NS-plasmid complex (Fig. 4D and E and Supplementary Fig. S12). In contrast, significantly lower amounts of the relaxed pUCsiteI and pUCsiteII plasmids were recovered from the complex (Fig. 4D and E and Supplementary Fig. S12), indicating that a strong bridge was formed between the replicon and HBS in both of the two plasmids. Recovery of the relaxed pUCsiteIII plasmid was not significantly different from pUC19 (Fig. 4D and E and Supplementary Fig. S12), suggesting a weaker bridge between the replicon and HBS in this plasmid. Together, our results reveal that positioning the HBS downstream of or opposite to the pMB1 replicon facilitates the formation of the DNA bridge within the plasmid.

We hypothesized that H-NS suppresses plasmid transfer by forming a DNA bridge within the incoming plasmid. Since HBS regions located opposite or downstream of the replicon are more likely to form DNA bridges than those located upstream of the replicon (Fig. 4E), we expected H-NS to exert a stronger suppressive effect on the transfer of pUCsiteI and pUCsiteII compared to pUC19 and pUCsiteIII. Consistent with this hypothesis, we observed that the transfer of pUCsiteI and pUCsiteII was more strongly suppressed by H-NS than that of pUC19 in WT *E. coli* (Fig. 4F). Notably, the transfer rate of pUCsiteIII was slightly but significantly lower than that of pUC19, suggesting that asymmetric bridging within the plasmid leads to differential impacts on suppression of plasmid transfer. In contrast, the transfer rates of all four plasmids were nearly identical in the Δ*hns* mutant, demonstrating that H-NS is essential for DNA-bridge-mediated repression of plasmid transfer (Fig. 4F). These findings reveal a positive correlation between H-NS-mediated bridging of the HBS and the replicon on the plasmid and the suppression of plasmid transfer, indicating that H-NS inhibits plasmid transfer by forming DNA bridges within the incoming plasmid. To further confirm that H-NS suppresses plasmid transfer through DNA bridging, we pre-incubated plasmids with H-NS under conditions conducive to *in vitro* DNA bridging and observed a significant reduction in TF in the Δ*hns* strain (Supplementary Fig. S13A). By contrast, pre-incubation with StpA had no significant effect on TF (Supplementary Fig. S13B). These results provide additional evidence that H-NS limits plasmid transfer by inducing plasmid structural changes through DNA bridging.

### Chromosomal expression of plasmid-derived replication initiation genes mitigates H-NS-mediated repression of plasmid transfer

**Figure 5.**
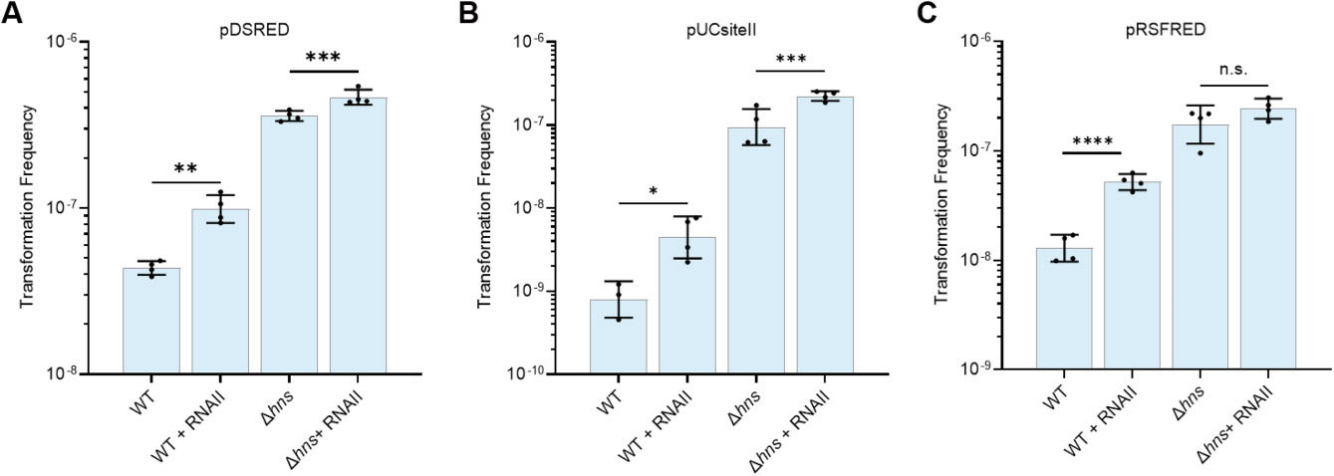
Effect of plasmid-encoded initiator on plasmid transfer. TFs of the WT, WT + RNAII, Δ*hns*, and Δ*hns* + RNAII strains were evaluated with ColE1 plasmids (**A**) pDSRED, (**B**) pUCsiteII, and (**C**) pRSFRED (*n* = 4). RNAII was constitutively expressed from chromosome. Data are shown as the geometric mean ± geometric SD. Statistical significance was assessed using a two-tailed Student’s *t*-test (* *P* ≤ .05, ** *P* ≤ .01, *** *P* ≤ .005, **** *P* ≤ .001, n.s. *P* > .05).

We hypothesized that H-NS suppresses plasmid transfer by forming an intramolecular DNA bridge that silences the transcription of plasmid-derived replication initiation genes, thereby limiting plasmid replication. To test this, we ectopically expressed the ColE1 plasmid-encoded RNAII, a replication primer required for the initiation of plasmid replication [77], by integrating its gene into the chromosome under a constitutive promoter. In WT, chromosomal expression of RNAII significantly increased TFs with pDSRED and pUCsiteII by 1.3- and 4.8-fold (Fig. 5A and B), respectively, indicating that transcriptional repression of RNAII is a limiting factor in plasmid transfer. In contrast, the same ectopic expression in a Δ*hns* mutant resulted in a much smaller increase in TFs (Fig. 5A and B). This trend was even more evident with pRSFRED, where RNAII expression significantly enhanced TFs in WT but had no significant effect in the Δ*hns* mutant (Fig. 5C). Chromosomal expression of RepA_pMUT2_, which synthesizes the primer RNA required for replication initiation of the ColE2 plasmid pMUT2 [96], led to a slight but significant increase in TF in the WT strain (Supplementary Fig. S14A). By contrast, chromosomal expression of RepA_pKD46_, the initiator required for replication of pSC101 plasmid pKD46 [97], had no significant effect on TF in either WT or Δ*hns* background (Supplementary Fig. S14B). These results suggest that the suppressive effect of H-NS on plasmid transfer through regulation of plasmid-encoded replication initiators varies depending on the plasmid.

### H-NS suppresses plasmid transfer during chemical transformation of *E. coli*

**Figure 6.**
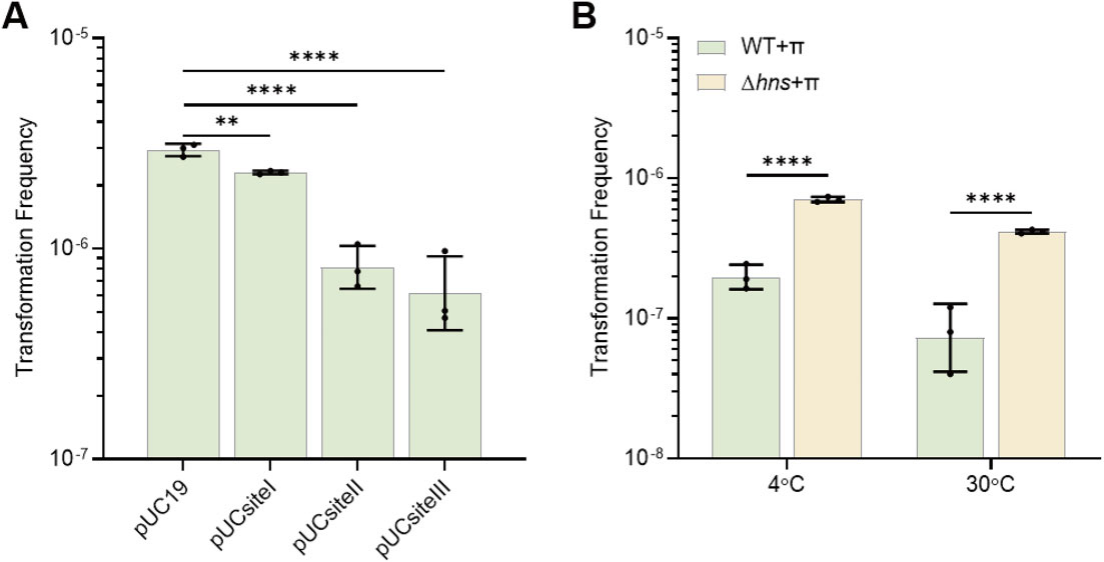
H-NS suppresses plasmid transfer during chemical transformation. (**A**) TFs of the WT BW25113 strain during chemical transformation with plasmids containing HBS. Plasmids pUC19, pUCsiteI, pUCsiteII, and pUCsiteIII were transferred into WT strain (*n* = 3). The chemically competent cells were incubated at 30°C for 30 min before heat shocking. (**B**) Plasmid pKD4 was transformed to Δ*hns* and WT expressing π replication initiator protein through chemical transformation (*n* = 3). Chemically competent cells were incubated at 4°C or 30°C for 30 min before heat shocking. Statistical significance was assessed using a two-tailed Student’s *t*-test (** *P* ≤ .01, **** *P* ≤ .001). Data are shown as the geometric mean ± geometric SD.

We investigated the potential impact of direct interactions between H-NS and plasmid on artificial transformation, considering that plasmid is presumed to enter chemically competent *E. coli* cells in dsDNA form [81]. However, we did not detect a significant positional effect of HBS on the transfer of pUC19 during electroporation and chemical transformation (Supplementary Fig. S15A). Given that H-NS tetramerization is significantly reduced at low temperatures [98], DNA-bridging mediated by H-NS may be weakened in competent cells incubated under such conditions. To evaluate the impact of temperature on H-NS activity, we incubated chemically competent cells at 30°C before heat shock at 42°C. Although TFs in WT and Δ*hns* were comparable with pUC19 as the donor plasmid (Fig. 6A and Supplementary Fig. S15B), we observed a significant inhibitory effect of H-NS on the transfer of pUC19 bearing HBS (Fig. 6A). In contrast, no significant difference in TFs was detected among plasmids in the Δ*hns* mutant (Supplementary Fig. S15B). Given the stronger inhibitory effect of H-NS on the transfer of pKD4 than on pUC19 during natural transformation (Fig. 1E), we further examined its impact on the transfer of pKD4 during artificial transformation. We found that H-NS also significantly reduced pKD4 transfer efficiency during chemical transformation (Fig. 6B). Notably, this inhibitory effect was further enhanced when chemically competent cells were pre-incubated at 30°C before heat shock at 42°C (Fig. 6B). These results provide additional support for the concept that H-NS broadly inhibits plasmid transfer. Notably, while only a slight suppression of pUCsiteIII by H-NS was observed during natural transformation (Fig. 4F), a strong suppression by H-NS was evident during chemical transformation (Fig. 6A), suggesting that H-NS-mediated DNA bridging may function differently in the two distinct transformation systems.

## Discussion

**Figure 7.**
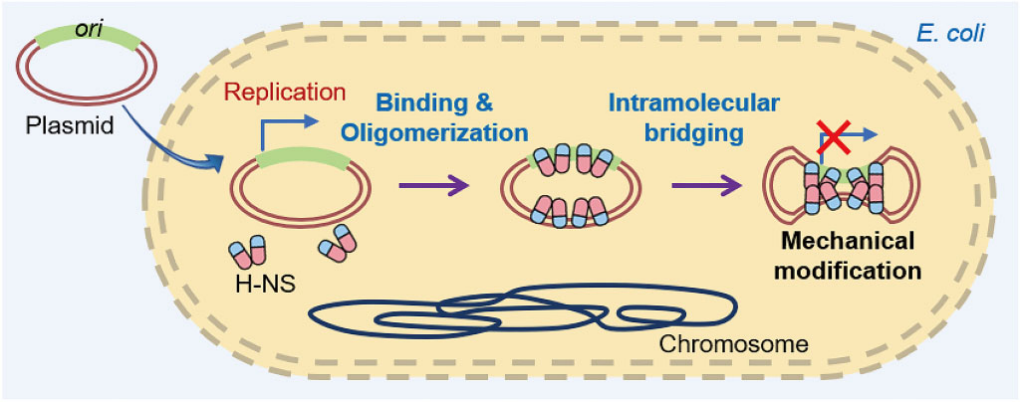
Schematic representation of the mechanism of prokaryotic defense via mechanical modification. Upon plasmid entry into the prokaryotic cell, H-NS recognizes, binds to, and oligomerizes along naked plasmid DNA, forming intramolecular DNA bridges. This mechanical modification of DNA prevents the plasmid from being inherited within the cell.

The principal finding of this work is the discovery that NAPs can directly participate in defense against invading plasmids by bridging DNA during natural transformation. While NAPs, including H-NS, have traditionally been recognized for their housekeeping functions during regular growth and survival in the absence of invading plasmids [39, 40], this work reveals their additional role in plasmid defense. Different from most dedicated defense systems, NAPs are expressed at a relatively high level [38, 40], and hence their surveillance against plasmids is likely constant, probably without inflicting strong physiological cost (Fig. 7).

In this study, we investigated the mechanism through which H-NS suppresses plasmid transfer. Our findings demonstrate that H-NS and many NAPs selectively and directly bind to dsDNA during the transformation process (Fig. 2). Notably, although Ler is known to antagonize H-NS in gene regulation, its ability to complement the transformation defect in the Δ*hns* mutant supports a model involving mechanical modulation rather than transcriptional regulation. Furthermore, we showed that the DNA-bridging ability of H-NS, as well as bridging of the plasmid origin that is mediated by H-NS, plays a crucial role in this process (Fig. 3). This work provides valuable insights into the mechanism by which H-NS represses plasmid transfer, representing a key step in understanding the broader mechanistic basis of host defense through mechanical modification of DNA. Considering the positional effects of the HBS relative to the replicon on both DNA bridging and plasmid transfer processes (Fig. 4), we speculate that H-NS may inhibit plasmid transfer by affecting plasmid replication and/or expression of antibiotic resistance genes (ARGs) from the plasmid. In the case of the ColE1 plasmid, ectopic expression of plasmid-derived RNAII effectively alleviated H-NS-mediated inhibition of plasmid transfer (Fig. 5), supporting this hypothesis. Importantly, the deletion of *hns* did not significantly reduce plasmid yield (Supplementary Fig. S16), suggesting that H-NS does not affect subsequent rounds of replication or expression of ARGs. It appears that cells recovered from transformation allow transcription of plasmid-encoded genes, which may counteract H-NS-mediated DNA bridging through transcription-driven supercoiling [99].

Interestingly, H-NS suppresses natural transformation but not conjugation (Fig. 2). During conjugation, incoming DNA is protected by host-encoded factors, with ssDNA-binding proteins safeguarding the ssDNA during transfer and UvrD facilitating its conversion to dsDNA [85]. This protein coating may shield the DNA from H-NS, preventing its binding. In contrast, natural transformation involves the uptake of naked plasmid dsDNA, which is more readily accessible to H-NS. The binding of H-NS to the plasmid may subsequently suppress the expression of genes essential for plasmid replication through the formation of intramolecular DNA bridges.

Of note, H-NS imposed stronger suppression on low-copy-number plasmids (Fig. 1). As replication progresses, the increasing copy number may generate more replication initiators, allowing plasmids to overcome H-NS-mediated inhibition. Although Pi protein was reported to be required for replication and copy number control of the R6K plasmid [100], induction of Pi protein expressed from the P_BAD_ promoter did not affect TF or copy number of the R6K plasmid pKD4 in the presence or absence of H-NS (Supplementary Fig. S1). Considering that R6K replication requires only low Pi levels, it is likely that leaky expression without an inducer might sustain sufficient Pi protein for replication.

Using TEM, we observed that H-NS induces lateral condensation across extensive regions of plasmid DNA (Fig. 4A), a phenomenon previously reported using AFM [92]. We speculate that this lateral condensation arises from H-NS-mediated DNA bridging. While earlier studies have demonstrated that H-NS binds and polymerizes along DNA to form a stiffened filament, they did not report compaction of linear DNA or chromosomal DNA [61]. Consistent with this, we did not detect compacted regions in linear DNA containing known HBSs (Supplementary Fig. S11). It is possible that, in plasmids, HBSs are brought into close physical proximity, thereby facilitating the formation of more compacted structures. Together with previous findings [61, 92], our results suggest that H-NS can compact plasmid DNA but does not similarly compact linear or chromosomal DNA. This selective compaction may represent a mechanism by which H-NS contributes to genome defense.

We considered the non-mutually exclusive possibility that H-NS may suppress plasmid transfer by modulating the transcription of host genes involved in natural transformation. Despite the reported regulation of RpoS by H-NS and its proposed role in transformation, our findings indicate that H-NS represses transformation through an RpoS-independent mechanism. Furthermore, disruption of other known transformation-related genes, along with a screen of membrane-associated proteins under H-NS control, revealed no involvement in H-NS-mediated repression of plasmid transfer. Although our results do not support a role for these targets, the possibility that H-NS acts through unidentified or condition-dependent host factors cannot be ruled out.

The mechanism of action of H-NS is characteristically different from known plasmid defense systems (Fig. 7). R-M systems distinguish foreign DNA through epigenetic modification and degrade non-self DNA with specific sequence motifs [3–5]. Mediated by small guide RNA, CRISPR–Cas systems recognize target nucleic acids, triggering specific or non-specific cleavage of nucleic acids [8–10]. Full-length Argonaute can cooperate with plasmid-unraveling helicase for target cleavage [34, 35]; short Argonaute can use nucleic acid guides to recognize and bind nucleic acid targets, eliciting programmed cell death due to NAD^+^ depletion and membrane disruption [11]. Gabija complex senses and executes nucleotide depletion and DNA cleavage for defense, triggering a cascade suicide effect [15, 16]. These systems mostly involve a sensing module and an effector module; the latter either performs target cleavage or induces cell death. In contrast, H-NS is a single-component system that functions by mechanically bridging invading DNA, probably inhibiting plasmid propagation by sequestering its replicon. One interesting aspect of analogy with other defense systems is the formation of protein assemblages such as the retron system [101], although the latter affects enzymatic rather than mechanical changes. This analogy underscores the novelty of the H-NS-mediated mechanical mode of action and highlights the potential for mechanical DNA modification to play a broader, fundamental role in bacterial defense. Given these insights, it would be interesting to further explore mechanical modification of DNA as a basic mechanism of defense across prokaryotes.

In conclusion, our study reveals a novel bacterial defense mechanism against plasmid transfer, wherein constitutive NAPs, specifically H-NS in *E. coli*, modulate the mechanical properties of foreign nucleic acids (Fig. 7). This mechanical modification of nucleic acids may represent an underexplored aspect of prokaryotic immunity against invading MGEs, highlighting a promising area for further investigation. Furthermore, given that plasmids are major vectors for the dissemination of ARGs [102], uncovering novel anti-plasmid mechanisms could provide new strategies for combating antibiotic resistance.

## Supplementary Material

gkaf928_Supplemental_File

## Data Availability

The data underlying this article are available in the article and in its online supplementary material.
