# Supplementary material for: Abundant bacterial nucleoid-associated protein H-NS limits plasmid transfer through mechanical modification of DNA
Source: Nucleic Acids Res. 2025 Sep 23;53(18):gkaf928. doi: 10.1093/nar/gkaf928 (PMC12455589; doi:10.1093/nar/gkaf928)
Supplement: gkaf928_Supplemental_File [file gkaf928_supplemental_file.docx]

**Abundant bacterial nucleoid-associated protein H-NS limits plasmid transfer through mechanical modification of DNA**

Mingyue Fei ^1, †^, Mengdie Fang ^2, 1, †^, Qi Zhou ^1^, Ziyan Chen ^1^, Mengxin Gong ^1^, Fabai Wu ^3^, Changfu Tian ^4, 5^, Dongchang Sun ^1*^

^1^ College of Biotechnology and Bioengineering, Zhejiang University of Technology, Hangzhou, Zhejiang, 310000, China

^2^ School of Laboratory Medicine and Bioengineering, Zhejiang Provincial People’s Hospital (Affiliated People’s Hospital), Hangzhou Medical College, Hangzhou, Zhejiang, 310000, China

^3^ School of Life Sciences, College of Science, Eastern Institute of Technology, Ningbo, Zhejiang, 315000, China

^4^ State Key Laboratory of Agrobiotechnology, and College of Biological Sciences, China Agricultural University, Beijing, 100000, China

^5^ MOA Key Laboratory of Soil Microbiology, and Rhizobium Research Center, China Agricultural University, Beijing, 100000, China

^†^ These authors contributed equally

*Corresponding author: Dongchang Sun, Email: [sundch@zjut.edu.cn](mailto:sundch@zjut.edu.cn)

**Supplementary methods**

**Plasmid yield quantification**

Strains were grown in LB broth with ampicillin (100 μg/mL) at 30°C. Plasmid was extracted from 2 mL of overnight cultures using the Axygen Plasmid Miniprep Kit. Plasmid concentrations were measured from 2 μL aliquots using a Nanovue spectrophotometer (Biotake). Plasmid yield was calculated by dividing the plasmid concentration by the OD_600_, and analyzed from at least three independent cultures for each strain.

**Protein purification**

The *E. coli* BL21 (DE3) strain was used as the host for the expression of H-NS and its variants with a 6×His-tag. When the culture was grown at 37°C to an OD_600_ of 0.4, expression of His_6_-H-NS or its variants was induced with 0.1 mM IPTG for 4 h. Total protein was isolated from *E. coli* cell pellets as previously described [1]. The 6×His-tagged protein was purified by using a tagged-protein purification kit (soluble protein) (Beijing ComWin Biotech Co., Ltd.). Purified His_6_-H-NS and its variants were separated by 12.5% SDS-PAGE.

**Western blot assay**

To quantify the expression level of RpoS in cells, total protein was extracted from cultures that had been incubated in M9 medium for 20 h. The protein samples were extracted by 12.5% SDS-PAGE and transferred to a 0.22 μm nitrocellulose transfer membrane (GVS). Immunoblotting was conducted using the anti-RpoS antibody (Biolegend Biotech Co., Ltd.) and anti-RpoB antibody (Biolegend Biotech Co., Ltd.) as primary antibodies. For detection, a horseradish peroxidase (HRP)-conjugated Goat anti-mouse IgG antibody (Biolegend Biotech Co., Ltd.) was utilized as the secondary antibody.

**Swarming motility assay**

The swarming motility assay was performed as previously described [2]. *E. coli* mutant strains were grown in LB medium at 37°C overnight. 2.5 µL samples were spotted on a 0.3% soft agar plate containing 1% tryptone and 0.25% NaCl. Plates were incubated at 37°C for 2-3 h and then transferred to room temperature overnight. The diameters of swarming zones were measured and then analyzed by averaging and normalizing to the BW25113 strain.

**Chemical transformation and electroporation assay**

*E. coli* BW25113 was grown in LB at 30°C until the OD_600_ reached 0.4-0.6. The cells were harvested and washed three times with deionized water or 100 mM CaCl_2_ for electrocompetent or chemical competent cells, respectively, after incubation on ice for 30 min. 50 µL of the competent cells were mixed with 1 µg of plasmid. For chemical transformation, the mixture was heat-shocked at 42°C for 90 s and then rapidly placed on ice for 2 min. For electroporation, the mixture was electroporated at the settings of 200 Ω, 25 µF and 1.8 kV. After transformation, the cells were supplemented with 950 µL LB and recovered at 30°C for 1 h. An appropriate amount of culture was spread on LB plates supplemented with ampicillin for screening transformants and antibiotic-free LB plates for counting viable cells respectively. Transformation frequency (TF) was defined as the ratio of transformants divided by the number of viable cells.

**Construction of *E. coli* mutants**

*E. coli* mutants were constructed by utilizing the λ-Red recombinase expressed by the temperature-sensitive plasmid pKD46 [3]. *E. coli* strains carrying pKD46 were grown at 30°C to an OD_600_ of 0.2, then 30 mM arabinose was added to the culture to induce the expression of the λ-Red system. The cells were made electrocompetent when the culture reached an OD_600_ of 0.4-0.6. The PCR product was generated with antibiotic resistance cassettes flanked by the homology extensions adjacent to the gene to be inactivated, followed by electroporation. Transformants were selected using plates with appropriate antibiotics. The resistance gene was eliminated by introducing the temperature-sensitive plasmid pCP20 into the mutant strain. All primers are listed in Supplementary Table S3.

To construct BW25113 ΔstpA and BW25113 Δhns mutants, the DNA fragments containing the FRT-flanked resistance gene (*cat* or *kan*) were amplified from the MC4100 ΔstpA::cat using the primer pair P_1_-P_2_ and from MC4100 Δhns::kan using the primer pair P_3_-P_4_, respectively. These fragments were then electroporated into competent cells.

To construct BW25113 ΔrpoS and BW25113 Δhns ΔrpoS mutants, the DNA fragment containing the FRT-flanked *cat* was amplified from the pKD3 plasmid using the primer pair P_76_-P_77_, and the upstream and downstream fragments of *rpoS* were amplified from the BW25113 genome using the primer pairs P_72_-P_73_ and P_74_-P_75_, respectively. The three DNA fragments were fused together and then electroporated into competent cells.

To construct BW25113 ΔmatP mutant, the DNA fragment containing the FRT-flanked *kan* was amplified from the pKD4 plasmid using the primer pair P_70_-P_71_, and the upstream and downstream fragments of *matP* were amplified from the BW25113 genome using the primer pairs P_66_-P_67_ and P_68_-P_69_, respectively. The three DNA fragments were fused together and then electroporated into competent cells.

To construct BW25113 hns^C^::kan mutant, the DNA fragment containing the FRT-flanked resistance gene (*kan*) was amplified from pKD4 with the primer pair P_25_-P_26_. The upstream and downstream fragments were amplified from the BW25113 genome using the primer pairs P_3_-P_23_ and P_4_-P_24_, respectively, followed by fusing with the FRT-flanked *kan* by using overlap PCR with the primer pair P_3_-P_4_. The fused DNA fragment (donor) was electroporated into competent cells. To construct *hns* variant complement strains, the *hns* variant fragments were amplified from pSU-P_BAD_-*hns* variant plasmids.

To examine the effect of H-NS bridging ‘inhibitor’ H-NS_1-58_ on plasmid transfer, we constructed the BW25113 Δ*lacZ::*HNS_1-58_ strain. This strain was constructed by amplifying the DNA fragment containing the FRT-flanked resistance gene (*kan*) from pKD4 with the primer pair P_70_-P_71_, the upstream and downstream fragments of the lacZ gene from the BW25113 genome using the primer pairs P_78_-P_79_ and P_80_-P_81_, respectively, and the DNA fragment containing H-NS_1-58_ expressing gene together with the promoter of *hns* from the *E. coli* BW25113 genome with the primer pair P_82_-P_83_, followed by fusing the four DNA fragments together and electroporating the fused DNA fragment into BW25113 competent cells.

To examine the effect of the plasmid-encoded initiator on plasmid transfer, we constructed the BW25113 RNAII and BW25113 RepA strains. RNAII and RepA were constitutively expressed on the chromosome. This strain was constructed by amplifying the DNA fragment containing the FRT-flanked resistance gene (*kan*) from pKD4 with the primer pair P_70_-P_71_, the upstream and downstream fragments of the insert site from the BW25113 genome using the primer pairs P_114_-P_115_ and P_118_-P_119_, respectively. The RNAII and RepA encoding genes were amplified from pUC19 and pMUT2 using the primer pairs P_116_-P_117_ and P_120_-P_121_, respectively. The DNA fragments were fused together and then electroporated into competent cells.

**PCR analysis for transformants**

The ΔstpA, Δhns and *hns* complement strains were verified by using colony PCR with the primer pair P_1_-P_2_ for ΔstpA and the primer pair P_3_-P_4_ for Δhns and *hns* complement strains. DNA sequencing was done by Qingke Biotech Co., Ltd.

**Construction of plasmids**

Plasmids pSURED and pRSFRED were constructed to evaluate the suppression of natural transformation by H-NS with different types of replicons. The fragments containing p15A replicon and pRSF replicon were separately amplified from pSU19 and pRSFDuet using the primer pairs P_7_-P_8_ and P_9_-P_10_, respectively. The linearized vector was obtained by PCR amplification of pDSRED with the primer pair P_5_-P_6_. The fragments and linearized vector were recombined using the ClonExpress II One Step Cloning Kit. Recombinant plasmids pSURED and pRSFRED were verified with the primer pair P_11_-P_12_.

Plasmids pHG101-motif and pHG101-motif* were constructed to investigate the potential effect of H-NS on transfer of ssDNA during conjugation. The DNA fragments (motif and motif*) were amplified from pGLO-motif and pGLO-motif*, respectively, using the primer pair P_15_-P_16_. The linearized vector was obtained by PCR amplification of pHG101 using the primer pair P_17_-P_18_. The fragment and linearized vector were recombined using the ClonExpress II One Step Cloning Kit. Recombinant plasmids pHG101-motif and pHG101-motif* were verified using the primer pair P_19_-P_20_.

The plasmid pMD20-*mob* was constructed to investigate the potential effect of H-NS on transfer of ssDNA during conjugation and natural transformation. The DNA fragment (*mob*) containing genes for mobilization was amplified from pHG101 with the primer pair P_27_-P_28_. The PCR product was ligated to T vector by using Mighty TA-cloning Kit.

To construct a series of plasmids expressing H-NS variants, we used the pSU-P_BAD_-*hns* as the template for site-directed mutagenesis. The primer pairs P_29_-P_30_, P_31_-P_32_, P_33_-P_34_, P_35_-P_36_ and P_37_-P_38_ were used to construct pSU-P_BAD_-*hns*^I70A^, pSU-P_BAD_-*hns*^I70C^, pSU-P_BAD_-*hns*^L70A^, pSU-P_BAD_-*hns*^L70C^ and pSU-P_BAD_-*hns*^G113C^ respectively. The primer pairs P_39_-P_40_ and P_41_-P_42_ were used to construct pSU-P_BAD_-*hns*^K83A, K87A, K89A, R90A, R93A^. The template plasmid was degraded with *Dpn*I and the PCR products were transformed into *E. coli* DH5α competent cells. Plasmids were isolated from transformants and confirmed by DNA sequencing, which was done by Qingke Biotech Co., Ltd.

To evaluate the effect of H-NS protein on R6K replicon plasmid transfer, we constructed the pSU-P_BAD_-π plasmid. The DNA fragment (*pir* gene encoding the π protein) was amplified from the *E. coli* WM3064 genome with the primer pair P_62_-P_63_. The linearized vector was obtained by PCR amplification of pSU-P_BAD_-*hns* using the primer pair P_64_-P_65_. The fragment and the linearized vector were recombined using the ClonExpress II One Step Cloning Kit.

Plasmids pUCsiteI, pUCsiteII, and pUCsiteIII were constructed to study the impact of HBS position on suppression of plasmid transfer by H-NS. The DNA fragment containing HBS was amplified from the BW25113 genome using the primer pair P_15_-P_16_. The linearized vectors were obtained by PCR amplification of pUC19 with the primer pairs P_46_-P_47_, P_48_-P_49_, and P_50_-P_51_, and recombined with the HBS fragment using the ClonExpress II One Step Cloning Kit, yielding plasmids pUCsiteI, pUCsiteII, and pUCsiteIII, respectively. All recombinant plasmids were verified by using colony PCR with the primer pair P_52_-P_53_.

Plasmids pSU-P_BAD_-*rok*, pSU-P_BAD_-*mvaT* and pSU-P_BAD_-*hns*_S.O._ were constructed to evaluate the effect of the heterologous xenogeneic silencers on repression of plasmid transfer during natural transformation. The linearized vector was obtained by PCR amplification of pSU-P_BAD_-*hns* with the primer pair P_54_-P_55_. The *rok* fragment was amplified from the *Bacillus subtilis* 168 genome using the primer pair P_56_-P_57_. The *mvaT* fragment was amplified from the *Pseudomonas aeruginosa* genome using the primer pair P_58_-P_59_. The *hns*_S.O._ fragment was amplified from the *Shewanella oneidensis* genome using the primer pair P_60_-P_61_. The fragments and the linearized vector were recombined using the ClonExpress II One Step Cloning Kit. The *mucR* from *Sinorhizobium fredii*, the *lsr2* from *Mycobacterium tuberculosis*, the *ler* from *E. coli* O157:H7 str. Sakai, and genes encoding H-NS family proteins on plasmids pEC14_35 (GenBank: AFC60903.1) and pMAS2027 (GenBank: ACV89896.1) from *E*. *coli* were chemically synthesized and cloned into linearized vector.

**Sequence of the biotin-labeled fragment I**

From 5’ to 3’:Biotin-TTCGGGAATGATTGTTATCAATGACGATAATAAGACCAATAACGGTTTATCCCTACTTAAGTAGGGAAGGTGCACAATGTACATCTTCTTTTAATTTCCCGGTATGAGATTTTATATTCACAGTATGAATATTTTATGTAATAAAATTCATGGTAATTATTATAACTAAAAGTTTCTTTAATAATAAAACGAATAACTTGCAGATTTGAAATGCATGCATTATTGTCTTTAAACAATTCAACACATCTTAATATATGTATAGGTTAATTGTATTAAACCAATGAATATATTTTTGCAGTGAATGTGATTATTGAATTAATTACGCCGTATTTTTTCTTTGTTTTTACCGATAACGGAAGTGTGCCGACGTATAGAAATGCAGGAGAAATGTCGGAGCATATGAAGGAGAACAA

**Sequence of the Cy5-labeled fragment II**

From 5’ to 3’:Cy5-ACAGAATCAGGGGATAACGCAGGAAAGAACATGTGAGCAAAAGGCCAGCAAAAGGCCAGGAACCGTAAAAAGGCCGCGTTGCTGGCGTTTTTCCATAGGCTCCGCCCCCCTGACGAGCATCACAAAAATCGACGCTCAAGTCAGAGGTGGCGAAACCCGACAGGACTATAAAGATACCAGGCGTTTCCCCCTGGAAGCTCCCTCGTGCGCTCTCCTGTTCCGACCCTGCCGCTTACCGGATACCTGTCCGCCTTTCTCCCTTCGGGAAGCGTGGCGCTTTCTCATAGCTCACGCTGTAGGTATCTCAGTTCGGTGTAGGTCGTTCGCTCCAAGCTGGGCTGTGTGCACGAACCCCCCGTTCAGCCCGACCGCTGCGCCTTATCCGGTAACTATCGTCTTGAGTCCAACCCGGTAAGACACGACTTATCGCCACTGGCAGCAGCCACTGGTAACAGGATTAGCAGAGCGAGGTATGTAGGCGGTGCTACAGAGTTCTTGAAGTGGTGGCCTAACTACGGCTACACTAGAAGAACAGTATTTGGTATCTGCGCTCTGCTGAAGCCAGTTACCTTCGGAAAAAGAGTTGGTAGCTCTTGATCCGGCAAACAAACCACCGCTGGTAGCGGTGGTTTTTTTGTTTGCAAGCAGCAGATTACGCGCAGAAAAAAAGGATCTCAAGAAGATCCTTTGATCTTTTCTACGGGGTCTGACGCTCAGTGGAACGAAAACTCACGTTAAGGGATTTTGGTCATGAGATTATCAAAAAGGATCTTCACCTAGATCCTTTTAAATTAAAAATGAAGTTTTAAATCAATCTAAAGTATATATGAGTAAACTTGGTCTGACAG

**Supplementary Tables**

**Supplementary Table S1 Strains used in this study**

| **Strain** | **Description** | **Sources/Reference** |
| --- | --- | --- |
| MC4100 | F^–^ λ^–^ *araD139* Δ(*argF-lac*)U169 *rpsL150* *relA* *deoC1 ptsF25 rbsR flbB5301* | Sun et al. [1] |
| MC4100 Δ*stpA* | MC4100 Δ*stpA::cm*, Cm^r^ | Sun et al. [1] |
| MC4100 Δ*hns* | MC4100 Δ*hns::kan*, Kan^r^ | Sun et al. [1] |
| BW25113 | F^–^ λ^–^ Δ(*araD-araB*)*567* Δ*lacZ4787* (::*rrnB*-3) *rph-1 hsdR514* Δ(*rhaD-rhaB*)*568* | Datsenko et al. [3] |
| BW25113 Δ*stpA* | BW25113 Δ*stpA::cat*, Cm^r^ | This study |
| BW25113 Δ*hns* | BW25113 Δ*hns::kan*, Kan^r^ | This study |
| BW25113 Δ*hha* | BW25113 Δ*hha::kan*, Kan^r^ | This study |
| BW25113 Δ*ydcS* | BW25113 Δ*ydcS::kan*, Kan^r^ | [4] |
| BW25113 Δ*ydcV* | BW25113 Δ*ydcV::kan*, Kan^r^ | [4] |
| BW25113 Δ*ompA* | BW25113 Δ*ompA::kan*, Kan^r^ | [5] |
| BW25113 Δ*hns* Δ*ydcS* | BW25113 Δ*hns::*FRT Δ*ydcS::*FRT | This study |
| BW25113 Δ*hns* Δ*ydcV* | BW25113 Δ*hns::*FRT Δ*ydcV::*FRT | This study |
| BW25113 Δ*hns* Δ*ompA* | BW25113 Δ*hns::*FRT Δ*ompA::*FRT | This study |
| BW25113 Δ*ydfJ* | BW25113 Δ*ydfJ::kan*, Kan^r^ | This study |
| BW25113 Δ*ynaI* | BW25113 Δ*ynaI::kan*, Kan^r^ | This study |
| BW25113 Δ*cusC* | BW25113 Δ*cusC::kan*, Kan^r^ | This study |
| BW25113 Δ*ybgQ* | BW25113 Δ*ybgQ::kan*, Kan^r^ | This study |
| BW25113 Δ*htrE* | BW25113 Δ*htrE::kan*, Kan^r^ | This study |
| BW25113 Δ*yehB* | BW25113 Δ*yehB::kan*, Kan^r^ | This study |
| BW25113 Δ*ompN* | BW25113 Δ*ompN::kan*, Kan^r^ | This study |
| BW25113 Δ*rpoS* | BW25113 Δ*rpoS::cat*, Cm^r^ | This study |
| BW25113 Δ*hns* Δ*rpoS* | BW25113 Δ*hns::kan* Δ*rpoS::cat*, Kan^r^ Cm^r^ | This study |
| BW25113 *hns^C^* | BW25113 *hns^C^*::*kan*, Kan^r^ | This study |
| BW25113 Δ*hns::hns*^I70A^ | BW25113 Δ*hns::hns*^I70A^::*kan*, Kan^r^ | This study |
| BW25113 Δ*hns::hns*^I70C^ | BW25113 Δ*hns::hns*^I70C^::*kan*, Kan^r^ | This study |
| BW25113 Δ*hns::hns*^L75A^ | BW25113 Δ*hns::hns*^L75A^::*kan*, Kan^r^ | This study |
| BW25113 Δ*hns::hns*^L75C^ | BW25113 Δ*hns::hns*^L75C^::*kan*, Kan^r^ | This study |
| BW25113 Δ*hns::hns*^G113C^ | BW25113 Δ*hns::hns*^G113C^::*kan*, Kan^r^ | This study |
| BW25113 Δ*hns::hns*^L-A5^ | BW25113 Δ*hns::hns*^K83A, K87A, K89A, R90A, R93A^::*kan*, Kan^r^ | This study |
| BW25113Δ*lacZ::*HNS_1-58_ | BW25113 Δ*lacZ::hns_1-58_::kan*, Kan^r^ | This study |
| BW25113 Δ*matP* | BW25113 Δ*matP*::*kan*, Kan^r^ | This study |
| BW25113 RNAII | BW25113 *::*RNAII*::kan*, Kan^r^ | This study |
| BW25113 Δ*hns* RNAII | BW25113 *::*RNAII*::kan* Δ*hns*, Kan^r^ | This study |
| BW25113 RepA_pMUT2_ | BW25113 *::*RepA_pMUT2_*::kan*, Kan^r^ | This study |
| BW25113 Δ*hns* RepA_pMUT2_ | BW25113 *::*RepA_pMUT2_*::kan* Δ*hns*, Kan^r^ | This study |
| BW25113 RepA_pKD46_ | BW25113 *::*RepA_pKD46_*::kan*, Kan^r^ | This study |
| BW25113 Δ*hns* RepA_pKD46_ | BW25113 *::*RepA_pKD46_*::kan* Δ*hns*, Kan^r^ | This study |
| WM3064 | Donor strain for conjugation: *thrB1004 pro thi rpsL hsdS lacZ*ΔM15 RP4-1360 Δ(*araBAD*)*567* Δ*dapA1341*::(*erm pir*(*wt*)) | Saltikov et al. [6] |
| *Bacillus subtilis* 168 | Wild-type | Yuan et al. [7] |
| *Pseudomonas aeruginosa* PAO1 | Wild-type | Lippa et al. [8] |
| *Shewanella oneidensi*s MR-1 | Wild-type | Yin et al. [9] |

**Supplementary Table S2 Plasmids used in this study**

| Plasmid | Inc Groups or Prototypes | Description | Size (bp) | Gene | Sources/Reference |
| --- | --- | --- | --- | --- | --- |
| pKD46 | pSC101 | Expressing λ-Red recombinase, *repA101* (TS) pSC101 *ori*, Amp^r^ | 6329 | 5 | Datsenko et al. [3] |
| pCP20 | pSC101 | Expressing FLP recombinase, *repA101* (TS) pSC101 *ori*, Cm^r^, Amp^r^ | 9332 | 5 | Datsenko et al. [3] |
| pSU19 | ColE1-like | p15A replicon, Cm^r^ | 2342 | 1 | Sun et al. [1] |
| pUC19 | ColE1-like | pMB1 replicon, Amp^r^ | 2686 | 2 | Norrander et al. [10] |
| pKD3 | IncX | R6K γ *ori*, Ts replicon, Cm^r^ | 2806 | 2 | Datsenko et al. [3] |
| pKD4 | IncX | R6K γ *ori*, Ts replicon, Kan^r^ | 3267 | 2 | Datsenko et al. [3] |
| pHG101 | IncP | RK2 replicon, Conjugation plasmid, Kan^r^ | 4158 | 4 | Wu et al. [11] |
| pGLO-P*_cas_*-*gfp* | ColE1-like | pGLO derivative, pMB1 replicon, *gfp* expressed with P*_cas_*, Amp^r^ | 4561 | 2 | Sun et al. [1] |
| pGLO-motif | ColE1-like | pGLO derivative, pMB1 replicon, with intact H-NS binding site, Amp^r^ | 4561 | 2 | Sun et al. [1] |
| pGLO-motif^*^ | ColE1-like | pGLO derivative, pMB1 replicon, with disrupted H-NS binding site, Amp^r^ | 4561 | 2 | This study |
| pSU-P_BAD_-*hns* | ColE1-like | pSU19 derivative, p15A replicon, H-NS expressed from P_BAD_, Cm^r^ | 3877 | 3 | Sun et al. |
| pSU-P_BAD_-*hns*^I70A^ | ColE1-like | pSU19 derivative, p15A replicon, H-NS^I70A^ expressed from P_BAD_, Cm^r^ | 3877 | 3 | This study |
| pSU-P_BAD_-*hns*^I70C^ | ColE1-like | pSU19 derivative, p15A replicon, H-NS^I70C^ expressed from P_BAD_, Cm^r^ | 3877 | 3 | This study |
| pSU-P_BAD_-*hns*^L75A^ | ColE1-like | pSU19 derivative, p15A replicon, H-NS^L75A^ expressed from P_BAD_, Cm^r^ | 3877 | 3 | This study |
| pSU-P_BAD_-*hns*^L75C^ | ColE1-like | pSU19 derivative, p15A replicon, H-NS^L75C^ expressed from P_BAD_, Cm^r^ | 3877 | 3 | This study |
| pSU-P_BAD_-*hns*^G113C^ | ColE1-like | pSU19 derivative, p15A replicon, H-NS^G113C^ expressed from P_BAD_, Cm^r^ | 3877 | 3 | This study |
| pSU-P_BAD_-*hns*^K83A, K87A, K89A, R90A, R93A^ | ColE1-like | pSU19 derivative, p15A replicon, H-NS^K83A, K87A, K89A, R90A, R93A^ expressed from P_BAD_, Cm^r^ | 3877 | 3 | This study |
| pRSFRED | ColE1-like | pDSRED derivative, pRSF replicon, expressing red fluorescent protein, Amp^r^ | 4188 | 3 | This study |
| pSURED | ColE1-like | pDSRED derivative, p15A replicon, expressing red fluorescent protein, Amp^r^ | 3893 | 3 | This study |
| pDSRED | ColE1-like | pUC19 derivative, pMB1 replicon, expressing red fluorescent protein, Amp^r^ | 4030 | 3 | Sun et al. [1] |
| pRSFDuet | ColE1-like | pRSF replicon, Kan^r^ | 3829 | 2 | Wuhan Transduction Biology Laboratory Co., Ltd. |
| pMUT2 | ColE2-like | *E. coli* Nissle 1917 endogenous cryptic plasmid pMUT2 derivate, ColE2 replicon, Cm^r^ | 5514 | 7 | This study |
| pMD20-*mob* | ColE1-like | pMB1 replicon, with *mob* gene, Amp^r^, Kan^r^ | 6428 | 5 | This study |
| pET-*hns* | ColE1-like | pET28a derivative, pMB1 replicon, H-NS expressed by T7/*lac* promoter, Kan^r^ | 5749 | 4 | Sun et al. [1] |
| pET-*stpA* | ColE1-like | pET28a derivative, pMB1 replicon, StpA expressed by T7/*lac* promoter, Kan^r^ | 5629 | 4 | Sun et al. [1] |
| pET-*hns*^I70C^ | ColE1-like | pET28a derivative, pMB1 replicon, His_6_-H-NS^I70C^ expressed by T7/*lac* promoter, Kan^r^ | 5749 | 4 | This study |
| pET-*hns*^L75C^ | ColE1-like | pET28a derivative, pMB1 replicon, His_6_-H-NS^L75C^ expressed by T7/*lac* promoter, Kan^r^ | 5749 | 4 | This study |
| pET-*hns*^G113C^ | ColE1-like | pET28a derivative, pMB1 replicon, His_6_-H-NS^G113C^ expressed by T7/*lac* promoter, Kan^r^ | 5749 | 4 | This study |
| pET-*hns*^K83A, K87A, K89A, R90A, R93A^ | ColE1-like | pET28a derivative, pMB1 replicon, His_6_-H-NS^L-A5^ expressed by T7/*lac* promoter, Kan^r^ | 5749 | 4 | This study |
| pUCsiteI | ColE1-like | pUC19 derivative, pMB1 replicon, with H-NS binding site, Amp^r^ | 3099 | 2 | This study |
| pUCsiteII | ColE1-like | pUC19 derivative, pMB1 replicon, with H-NS binding site, Amp^r^ | 3099 | 2 | This study |
| pUCsiteIII | ColE1-like | pUC19 derivative, pMB1 replicon, with H-NS binding site, Amp^r^ | 3099 | 2 | This study |
| pSU-P_BAD_-*rok* | ColE1-like | pSU19 derivative, p15A replicon, expressing Rok from P_BAD_, Cm^r^ | 4039 | 3 | This study |
| pSU-P_BAD_-*mvaT* | ColE1-like | pSU19 derivative, p15A replicon, expressing MvaT from P_BAD_, Cm^r^ | 3838 | 3 | This study |
| pSU-P_BAD_-*hns*_S.O._ | ColE1-like | pSU19 derivative, p15A replicon, expressing H-NS_S.O._ from P_BAD_, Cm^r^ | 3856 | 3 | This study |
| pSU-P_BAD_-*mucR* | ColE1-like | pSU19 derivative, p15A replicon, expressing MucR from P_BAD_, Cm^r^ | 3895 | 3 | This study |
| pSU-P_BAD_-*lsr2* | ColE1-like | pSU19 derivative, p15A replicon, expressing Lsr2 from P_BAD_, Cm^r^ | 3802 | 3 | This study |
| pSU-P_BAD_-*ler* | ColE1-like | pSU19 derivative, p15A replicon, expressing Ler from P_BAD_, Cm^r^ | 3835 | 3 | This study |
| pSU-P_BAD_-*hns*_pEC_ | ColE1-like | pSU19 derivative, p15A replicon, expressing H-NS like-protein pEC14_35 from P_BAD_, Cm^r^ | 3919 | 3 | This study |
| pSU-P_BAD_-*hns*_pMAS_ | ColE1-like | pSU19 derivative, p15A replicon, expressing H-NS-like protein pMAS2027 from P_BAD_, Cm^r^ | 3925 | 3 | This study |
| pSU-P_BAD_-π | ColE1-like | pSU19 derivative, p15A replicon, expressing π protein from P_BAD_, Cm^r^ | 4381 | 3 | This study |
| pET-HNS_1-58_ | ColE1-like | pET28a derivative, His_6_-H-NS_1-58_ expressed by T7/*lac* promoter, Kan^r^ | 5512 | 4 | This study |

**Supplementary Table S3** **Oligonucleotides used in this study**

|  | Oligonucleotides | Sequences (5’-3’) |
| --- | --- | --- |
| P_1_ | Δ*stpA*-F | TAATCTGATGCCAGTCTGCG |
| P_2_ | Δ*stpA*-R | ATTTTAGCGGAGCCTGCC |
| P_3_ | Δ*hns*-F | AGTAAGTATTGGCTGCCAAT |
| P_4_ | Δ*hns*-R | AGGAACCAGGATGTTGCCGG |
| P_5_ | pDSRED-F1 | CATGTTCTTTCCTGCGTTATC |
| P_6_ | pDSRED-R1 | CTTGGTCTGACAGTTACCAA |
| P_7_ | Ori-pSU19-F | TGGTAACTGTCAGACCAAGGGCAGTTATTGGTGCCTAGA |
| P_8_ | Ori-pSU19-R | AACGCAGGAAAGAACATGCGGAGATTTCCTGGAAGATG |
| P_9_ | Ori-pRSFDuet-F | TGGTAACTGTCAGACCAAGTAGGCATGCAGCGCTCTT |
| P_10_ | Ori-pRSFDuet-R | AACGCAGGAAAGAACATGGCGCAACGCAATTAATGTAAGT |
| P_11_ | JC Ori-F | GTCTCGCGGTATCATTGCA |
| P_12_ | JC Ori-R | TCTTCCGCTTCCTCGCTC |
| P_13_ | pSUP_BAD_HNS-F | GCATGGATGAGCTCTACAAATAAGAATTCGAGCTCGGTACC |
| P_14_ | pSUP_BAD_HNS-R | TTCTCCTTTGCTAGCCATTTGCTTGATCAGGAAATCG |
| P_15_ | P*_cas_*-F | TTCGGGAATGATTGTTATCAATGACG |
| P_16_ | P*_cas_*-R | TTGTTCTCCTTCATATGCTCCG |
| P_17_ | pHG101-F | GAGCATATGAAGGAGAACAACTGAATTCAGTCGACCCTCG |
| P_18_ | pHG101-R | TGATAACAATCATTCCCGAAGTACCACCCGGGGCTAGCA |
| P_19_ | JC101-P*_cas_*-F | TGAGCAAACTGGCCTCAGG |
| P_20_ | JC101-P*_cas_*-R | TGACGAGTTCTTCTGAGCG |
| P_21_ | HBS-F | TAATTATTATAACTAAAAGTTTCTTTAATAATAAAACGAATAACTTGCAGATTTGAAAT |
| P_22_ | HBS-R | ATTTCAAATCTGCAAGTTATTCGTTTTATTATTAAAGAAACTTTTAGTTATAATAATTA |
| P_23_ | UphnsFRT-R | GAAGCAGCTCCAGCCTACACTTATTGCTTGATCAGGAAATCGTC |
| P_24_ | hnsFRTdown-F | GGAGGATATTCATATGGACCTCTTTTGTAGATTGCACTTGCTTAAAATCC |
| P_25_ | Kan-F | GTGTAGGCTGGAGCTGCTTC |
| P_26_ | Kan-R | GGTCCATATGAATATCCTCC |
| P_27_ | Mob-F | GGTCACGACTTTGCGAAG |
| P_28_ | Mob-R | CACTTGGGTTGCGCAG |
| P_29_ | hnsI70A-F | CGCTGACGGTGCTGACCCGAACGAAC |
| P_30_ | hnsI70A-R | CGTTCGGGTCAGCACCGTCAGCGATC |
| P_31_ | hnsI70C-F | CGCTGACGGTTGTGACCCGAACGAAC |
| P_32_ | hnsI70C-R | CGTTCGGGTCACAACCGTCAGCGATC |
| P_33_ | hnsL75A-F | GACCCGAACGAAGCTCTGAATAGCCTTGC |
| P_34_ | hnsL75A-R | GCAAGGCTATTCAGAGCTTCGTTCGGGTC |
| P_35_ | hnsL75C-F | GACCCGAACGAATGTCTGAATAGCCTTGC |
| P_36_ | hnsL75C-R | GCAAGGCTATTCAGACATTCGTTCGGGTC |
| P_37_ | hnsG113C-F | GACTGGCCAATGCCGTACTCCAGCTG |
| P_38_ | hnsG113C-R | CTGGAGTACGGCATTGGCCAGTCCAG |
| P_39_ | hnsLA5-1F | CTGCCGTTGCATCTGGCACC |
| P_40_ | hnsLA5-1R | GGTGCCAGATGCAACGGCAG |
| P_41_ | hnsLA5-2F | TCTGGCACCGCAGCTGCAGCTGCTCAGGCTCCGGCAAAATATAGCTACG |
| P_42_ | hnsLA5-2R | CGTAGCTATATTTTGCCGGAGCCTGAGCAGCTGCAGCTGCGGTGCCAGA |
| P_43_ | BioP*_cas_*-F | Biotin-TTCGGGAATGATTGTTATCAATGACG |
| P_44_ | Cy5Ori-F | Cy5-ACAGAATCAGGGGATAACGC |
| P_45_ | Ori-R | CTGTCAGACCAAGTTTACTC |
| P_46_ | HBSsite I-F | CGGAGCATATGAAGGAGAACAAGCCTTTGAGTGAGCTGATAC |
| P_47_ | HBSsiteI -R | CGTCATTGATAACAATCATTCCCGAATGCTGGCGTTTTTCCATAG |
| P_48_ | HBSsite II-F | CGGAGCATATGAAGGAGAACAATGGCACTTTTCGGGGAAAT |
| P_49_ | HNSsite II-R | CGTCATTGATAACAATCATTCCCGAAAGCTTGTCTGTAAGCGGAT |
| P_50_ | HBSsite III-F | CGGAGCATATGAAGGAGAACAAAGACCCCGTAGAAAAGATCA |
| P_51_ | HBSsite III-R | CGTCATTGATAACAATCATTCCCGAAGTCTGACAGTTACCAATGCT |
| P_52_ | JCP*_cas_*-F | GCAGATTTGAAATGCATGCA |
| P_53_ | JCpUC-ORI-R | AGGACTATAAAGATACCAGGCG |
| P_54_ | pSUP_BAD_-F | GAATTCGAGCTCGGTACCC |
| P_55_ | pSUP_BAD_-R | ATGTATATCTCCTTCTTAAAG |
| P_56_ | pSURok-F | CTTTAAGAAGGAGATATACATATGTTTAATGAAAGAGAAGCTTTGC |
| P_57_ | pSURok-R | CGGGTACCGAGCTCGAATTCTTATTCGTTTGCTGATTCTGCAG |
| P_58_ | pSUMvaT-F | CTTTAAGAAGGAGATATACATATGTCCCTGATCAACGAATATC |
| P_59_ | pSUMvaT-R | CGGGTACCGAGCTCGAATTCTTAGCCGAGCAGGGTGGCC |
| P_60_ | pSUHNS_S.O._-F | CTTTAAGAAGGAGATATACATATGAGCGAATTTTTAGAAATATTAACTCACG |
| P_61_ | pSUHNS_S.O._-R | CGGGTACCGAGCTCGAATTCTTAGATTAAGAAATCATCCATAGAACGGC |
| P_62_ | π-F | ATGAGACTCAAGGTCATGAT |
| P_63_ | π-R | CCCCTTAGCTTTTTTGGG |
| P_64_ | pSUP_BAD_π-F | CCTCCCAAAAAAGCTAAGGGGTAAGAATTCGAGCTCGGTACC |
| P_65_ | pSUP_BAD_π-R | CATCATGACCTTGAGTCTCATATGTATATCTCCTTCTTAAAGTTAAAC |
| P_66_ | matp H1-F | CGACAACTTGCGTTAAAGTC |
| P_67_ | matp H1-R | GAAGCAGCTCCAGCCTACACAGCGATCTCAATGTTACCGTG |
| P_68_ | matp H2-F | GGAGGATATTCATATGGACCCTGACGAAAGTCAGTTCAATTTAC |
| P_69_ | matp H2-R | GAAGCAGCTCCAGTAGTTGC |
| P_70_ | FRT Kan-F | GTGTAGGCTGGAGCTGCTTC |
| P_71_ | FRT Kan-R | GGTCCATATGAATATCCTCC |
| P_72_ | RpoS H1-F | GCCTGGATTACTGGCAACG |
| P_73_ | RposSH1-R | AAGCAGCTCCAGCCTACAAAGGTGGCTCCTACCCG |
| P_74_ | RpoS H2-F | TAAGGAGGATATTCATATGTAAGCATCTGTCAGAAAGGCC |
| P_75_ | RpoS H2-R | CTGGAACAGATGCAACTGGC |
| P_76_ | FRT Cm-F | TGTAGGCTGGAGCTGC |
| P_77_ | FRT Cm-R | ATATGAATATCCTCCTTAGTTCC |
| P_78_ | lacZ H1-F | GAACGATGAGCCAATAATACG |
| P_79_ | lacZ H1-R | GTGAATAGTTACCAGCAGTCTAATAACCGGGCAGGCCATG |
| P_80_ | lacZ H2-F | GGAGGATATTCATATGGACCTGCGCTCACTGCCCGCTTTC |
| P_81_ | lacZ H2-R | CAGCAAATCGCGCTGTTAGC |
| P_82_ | PHNS_1-58_-F | GACTGCTGGTAACTATTCAC |
| P_83_ | PHNS_1-58_-R | GAAGCAGCTCCAGCCTACACCAGTTTACGAGTGCGCTCTTC |
| P_84_ | ydfJ H1-F | GCTACTTCCACTACAGCATC |
| P_85_ | ydfJ H1-R | GAAGCAGCTCCAGCCTACACAATTCACAGGTGTTTTTTCCCATC |
| P_86_ | ydfJ H2-F | GGAGGATATTCATATGGACCACCCTTAATCCCTTCTCTTACC |
| P_87_ | ydfJ H2-R | ATACGCCAGGAATGAATGAC |
| P_88_ | ynaI H1-F | GCTGGCTTTCATGTATTCGATG |
| P_89_ | ynaI H1-R | GAAGCAGCTCCAGCCTACACTGTTTTATCAGGCTCCTCCAG |
| P_90_ | ynaI H2-F | GGAGGATATTCATATGGACCATCAGACATCACTGGCGTTATG |
| P_91_ | ynaI H2-R | GGTCGTGGTACCGAATACCC |
| P_92_ | cusC H1-F | ATCCTGCTCGGCAAAATGAA |
| P_93_ | cusC H1-R | GAAGCAGCTCCAGCCTACACTATGCCGCCAACTTTACTCG |
| P_94_ | cusC H2-F | GGAGGATATTCATATGGACCGCTCACCCCAGTCACTTAC |
| P_95_ | cusC H2-R | ATACTCACTCTGCGCTTCCA |
| P_96_ | ybgQ H1-F | CGTTCATATGGCGGGTTGAT |
| P_97_ | ybgQ H1-R | GAAGCAGCTCCAGCCTACACGTAGGTTGCGTTAGCGGTTA |
| P_98_ | ybgQ H2-F | GGAGGATATTCATATGGACCCATCAGCTTGGGTTGCAATG |
| P_99_ | ybgQ H2-R | TGCCGTTGGTATTGTTGTCA |
| P_100_ | htrE H1-F | CTGGATGCAACGACAAAATC |
| P_101_ | htrE H1-R | GAAGCAGCTCCAGCCTACACGCTATGCTTCCTGCGGAATTATATC |
| P_102_ | htrE H2-F | GGAGGATATTCATATGGACCCTACAAGGGACGTCAAATG |
| P_103_ | htrE H2-R | GCGACAGCACTTGCTTTATC |
| P_104_ | yehB H1-F | CGATAGCCAGTAGCAACGAT |
| P_105_ | yehB H1-R | GAAGCAGCTCCAGCCTACACGGTCAGATGCCAGTTATTTGC |
| P_106_ | yehB H2-F | GGAGGATATTCATATGGACCTGATGGTTATGCCTGTGAGC |
| P_107_ | yehB H2-R | GTAAGTTTTGACTTCGCCGC |
| P_108_ | ompN H1-F | AACGTCAACATTCTGGTGCT |
| P_109_ | ompN H1-R | GAAGCAGCTCCAGCCTACACCCCATTTCGGGGAGTGAAAAC |
| P_110_ | ompN H2-F | GGAGGATATTCATATGGACCGTGTCATCTGGCGTTACACT |
| P_111_ | ompN H2-R | TCGTAGCTTATTGTTGTCGCT |
| P_112_ | pUC-F | ACAGCTTGTCTGTAAGCGGA |
| P_113_ | pUC-R | GACCGTCTCCGGGAGCTGCA |
| P_114_ | RNAII H1-F | GCGCAGGTTAATGCTATCAA |
| P_115_ | RNAII H1-R | GACGGATTCTGGCAAATAGAC |
| P_116_ | RNAII-F | GTCCTAGGTACAGTGCTAGCCTTGCAAACAAAAAAACCACC |
| P_117_ | RNAII-R | CACAATCGCTCAAGACGTGAACGCAGGAAAGAACATGT |
| P_118_ | RNAII H2-F | CCCATGTCAGCCGTTAAGTGGACTTATAATCGCCAGAACGG |
| P_119_ | RNAII H2-R | CAGGCACTGCATATTGCAC |
| P_120_ | RepA_pMUT2_-F | GTCTATTTGCCAGAATCCGTCTTGACGGCTAGCTCAGTC |
| P_121_ | RepA_pMUT2_-R | CACAATCGCTCAAGACGTGTTATTTGGCGCTGTACTTG |
| P_122_ | RepA_pKD46_-F | GTCTATTTGCCAGAATCCGTCTTGACGGCTAGCTCAGTC |
| P_122_ | RepA_pKD46_-R | CACAATCGCTCAAGACGTGCTGTTTTATCAGACCGCTTCT |

**Supplementary Figures**

**~~
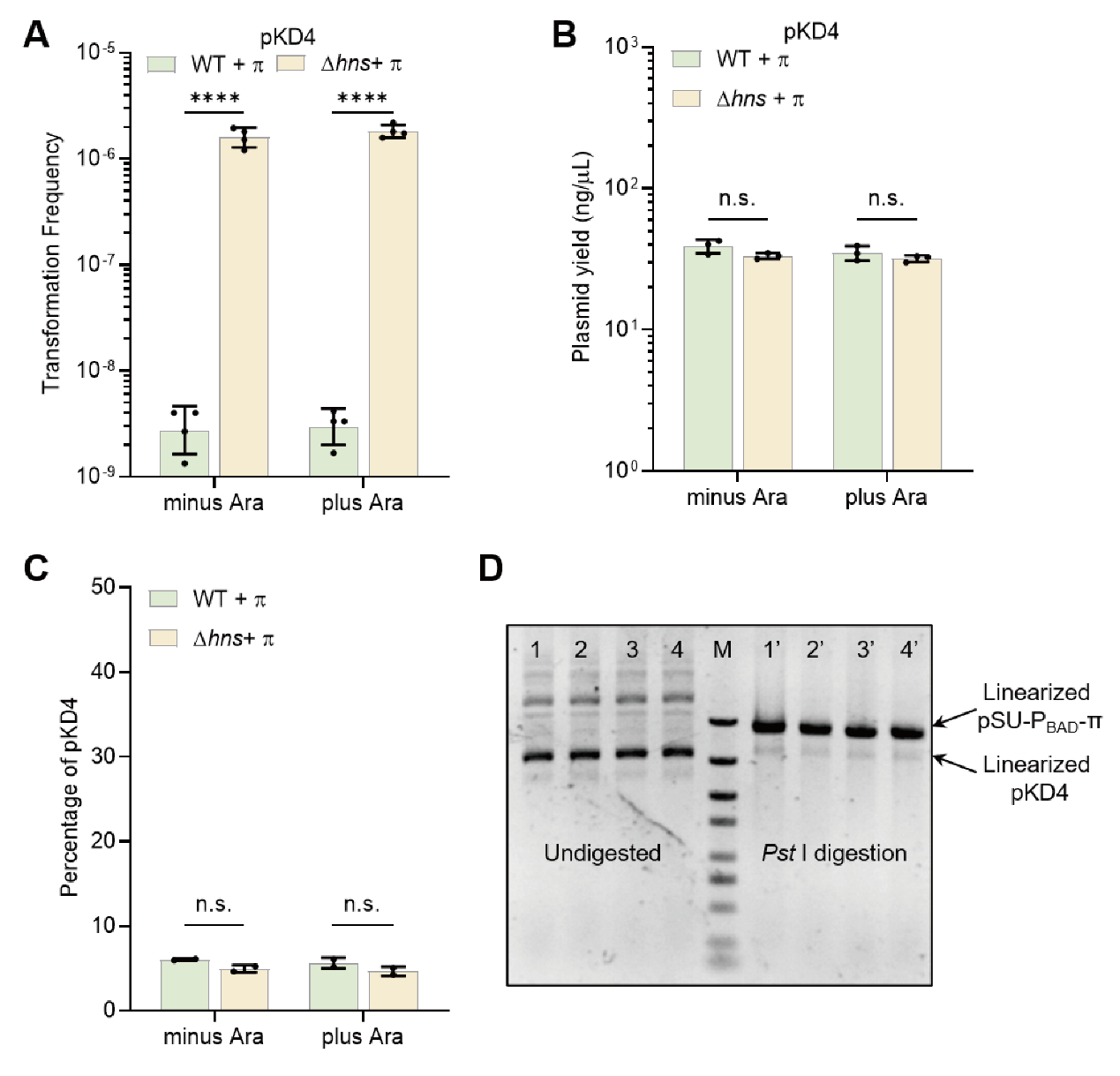
~~**

**Supplementary Figure S1.** Effect of π protein on pKD4 plasmid transfer. (**A**) Plasmid pKD4 was transformed into Δ*hns* and WT strains that contain pSU-P_BAD_-π (*n* = 4). The π replication initiator protein was induced without (minus Ara) or with 20 mM arabinose (plus Ara). (**B**) Plasmid yields of pKD4 in the Δ*hns* and WT strains containing pSU-P_BAD_-π. The π protein was induced without (minus Ara) or with 20 mM arabinose (plus Ara). Plasmid yield was calculated by dividing the plasmid concentration by OD_600_ (*n* = 4). (**C**) Percentage of pKD4 in total plasmid extracted from strains in (**B**). Percentage of pKD4 was quantified via the intensity of the corresponding band in (**D**) using ImageJ (*n* = 2). (**D**) Representative agarose gel electrophoresis of the plasmids before or after *Pst* I digestion. The extracted plasmids (channels 1’ to 4’) were digested with *Pst* I. Channels 1 to 4 were the undigested control groups. The pSU-P_BAD_-π plasmid (4381 bp) and pKD4 (3267 bp) had a *Pst* I cleavage site. M: 5000 DL marker (from top to bottom: 5 kb, 3 kb, 2 kb, 1.5 kb, 1 kb, 750 bp, 500 bp, 250 bp, 100 bp). Channels 1 and 1’ were plasmids from WT + π without Ara, Channels 2 and 2’ were plasmids from WT + π with Ara, Channels 3 and 3’ were plasmids from Δ*hns* + π without Ara, Channels 4 and 4’ were plasmids from Δ*hns* + π with Ara. Statistical significance was assessed using a two-tailed Student’s *t*-test (n.s. *p* > 0.05). Data are shown as the geometric mean ± geometric SD.

**
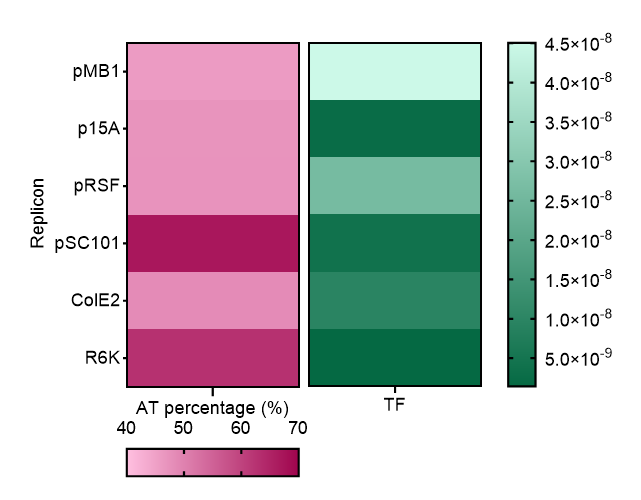
**

**Supplementary Figure S2.** Heatmap visualization of AT percentage of replicon and corresponding transformation frequency (TF). Correlation between AT percentage of plasmid replicon (red rectangle on the left) and TF (green rectangle on the right).

**
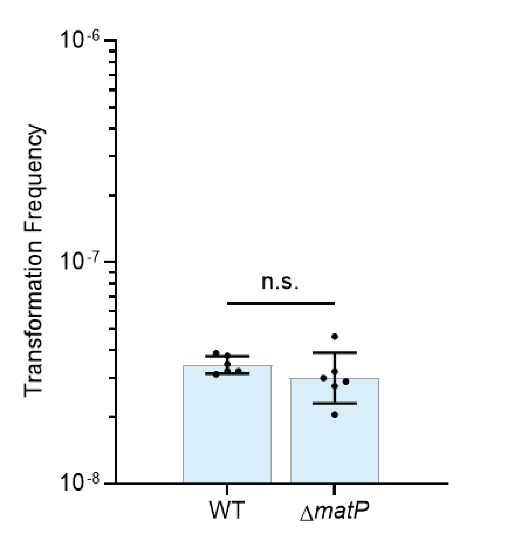
**

**Supplementary Figure S3.** Effect of MatP on natural transformation. TFs of the Δ*matP* and wild-type (WT) strain (*n* = 6). The method for constructing the mutant strain is described in Supplementary methods. Statistical significance was assessed using a two-tailed Student’s *t*-test (n.s. *p* > 0.05). Data are shown as the geometric mean ± geometric SD.

**
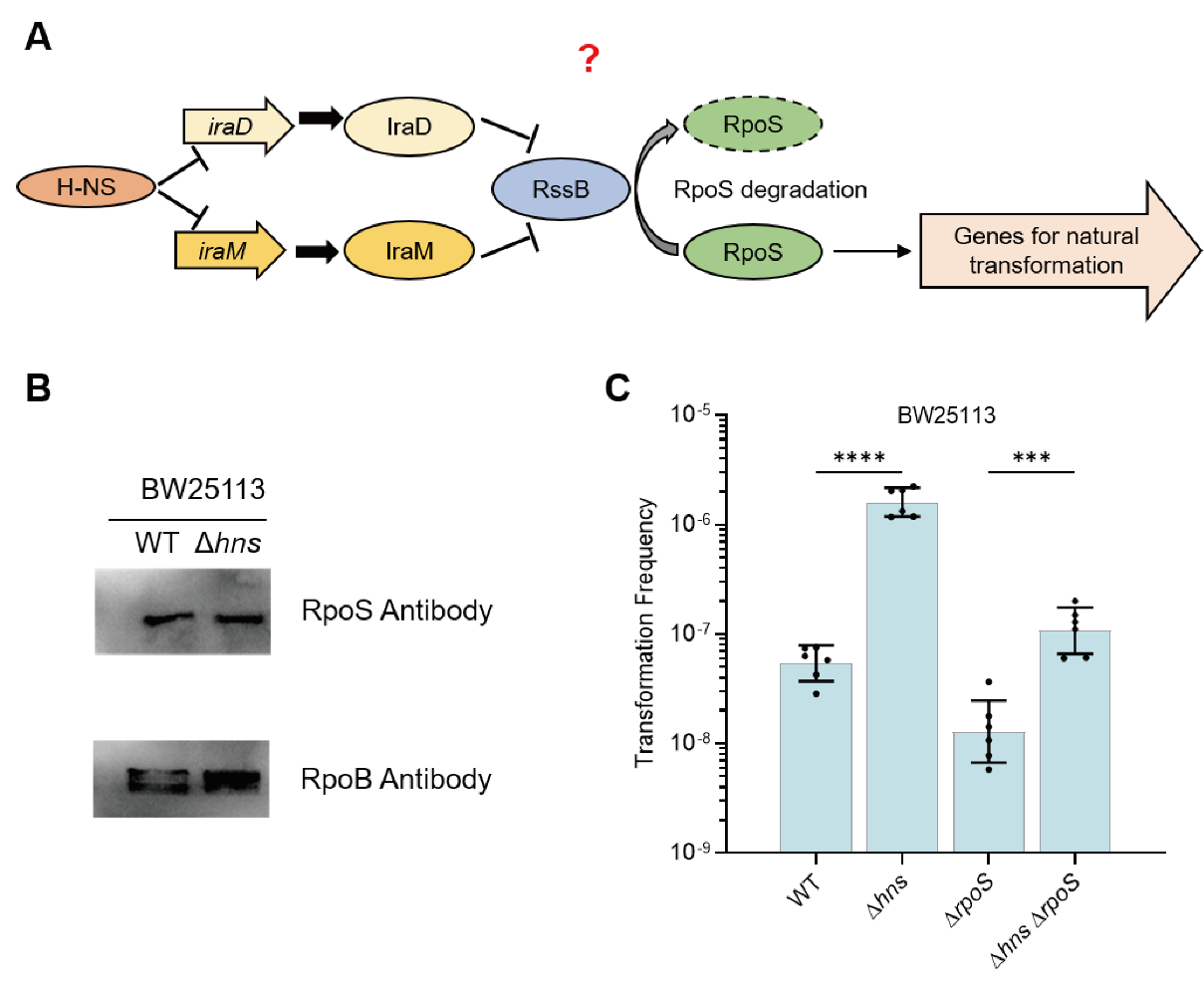
**

**Supplementary Figure S4.** H-NS suppresses plasmid transfer independent of RpoS during natural transformation. (**A**) Postulated mechanism of H-NS regulation of natural transformation through RpoS. H-NS affects RpoS which regulates competent relative genes. (**B**) Western blot for detecting RpoS protein level in the WT and Δ*hns* strains cultured in M9 medium for 20 h, with RpoB serving as the internal control. Strains cultured in M9 medium for 20 h are used for Western blot assay. (**C**) TFs of BW25113 and its *rpoS* and *hns* single- and double-deletion mutants with pDSRED as the donor plasmid (*n* = 6). The method for constructing the mutant strain is described in Supplementary methods. Statistical significance was assessed using a two-tailed Student’s *t*-test (*** *p* ≤ 0.005, **** *p* ≤ 0.001). Data are shown as the geometric mean ± geometric SD.

**
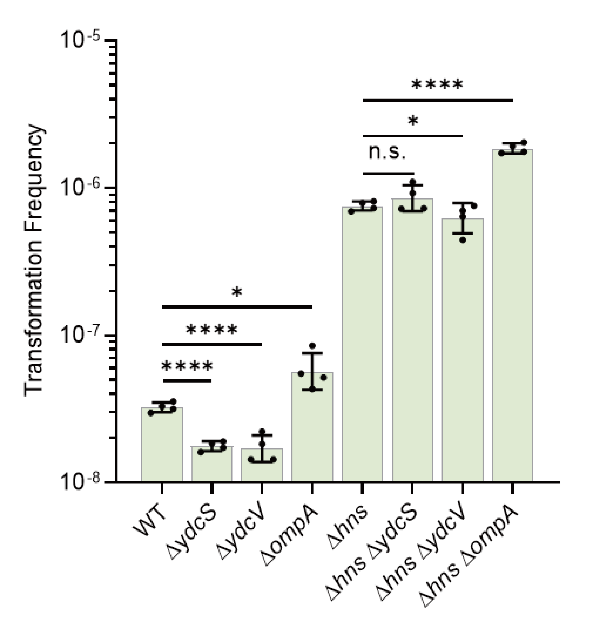
**

**Supplementary Figure S5.** Effect of *hns* inactivation on natural transformation of *E. coli* defective in genes involved in plasmid transfer. TFs of BW25113 and *ydcS*, *ydcV,* and *ompA* mutant strains with pDSRED as the donor plasmid (*n* = 4). Statistical significance was assessed using a two-tailed Student’s *t*-test (* *p* ≤ 0.05, **** *p* ≤ 0.001, n.s. *p* > 0.05). Data are shown as the geometric mean ± geometric SD.

**
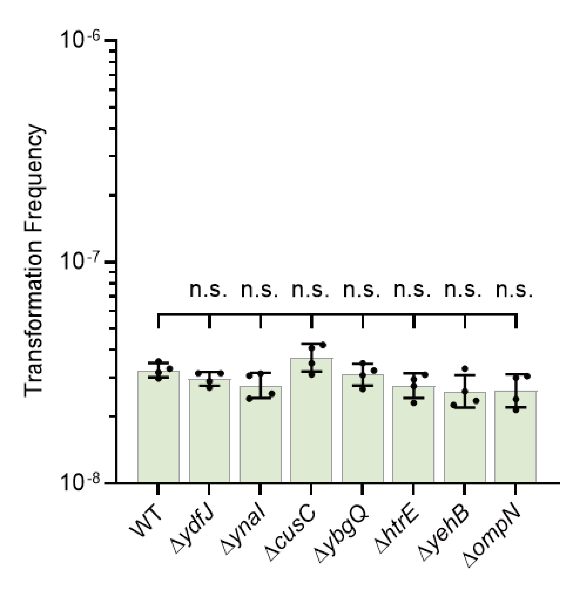
**

**Supplementary Figure S6.** Effect of inactivation of H-NS-regulated membrane protein-related genes on natural transformation (*n* = 4). Statistical significance was assessed using a two-tailed Student’s *t*-test (n.s. *p* > 0.05). Data are shown as the geometric mean ± geometric SD.

**
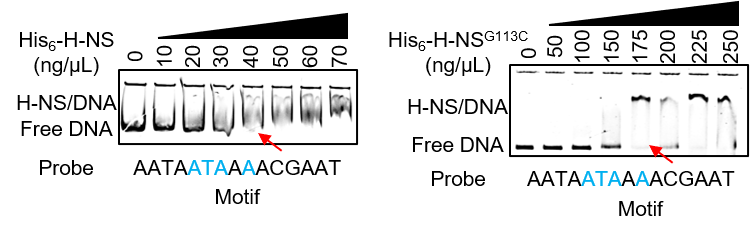
**

**Supplementary Figure S7.** Binding affinity between H-NS protein and probe DNA. Electrophoretic mobility shift assay with probes containing the intact motif was performed using His_6_-H-NS and His_6_-H-NS^G113C^. The sample was separated with 5% native polyacrylamide gels. Probe concentration: 1 ng/μL. The numbers above the lanes represent the protein concentrations used. The red arrows mark the minimum protein concentration required for detectable binding to free DNA.


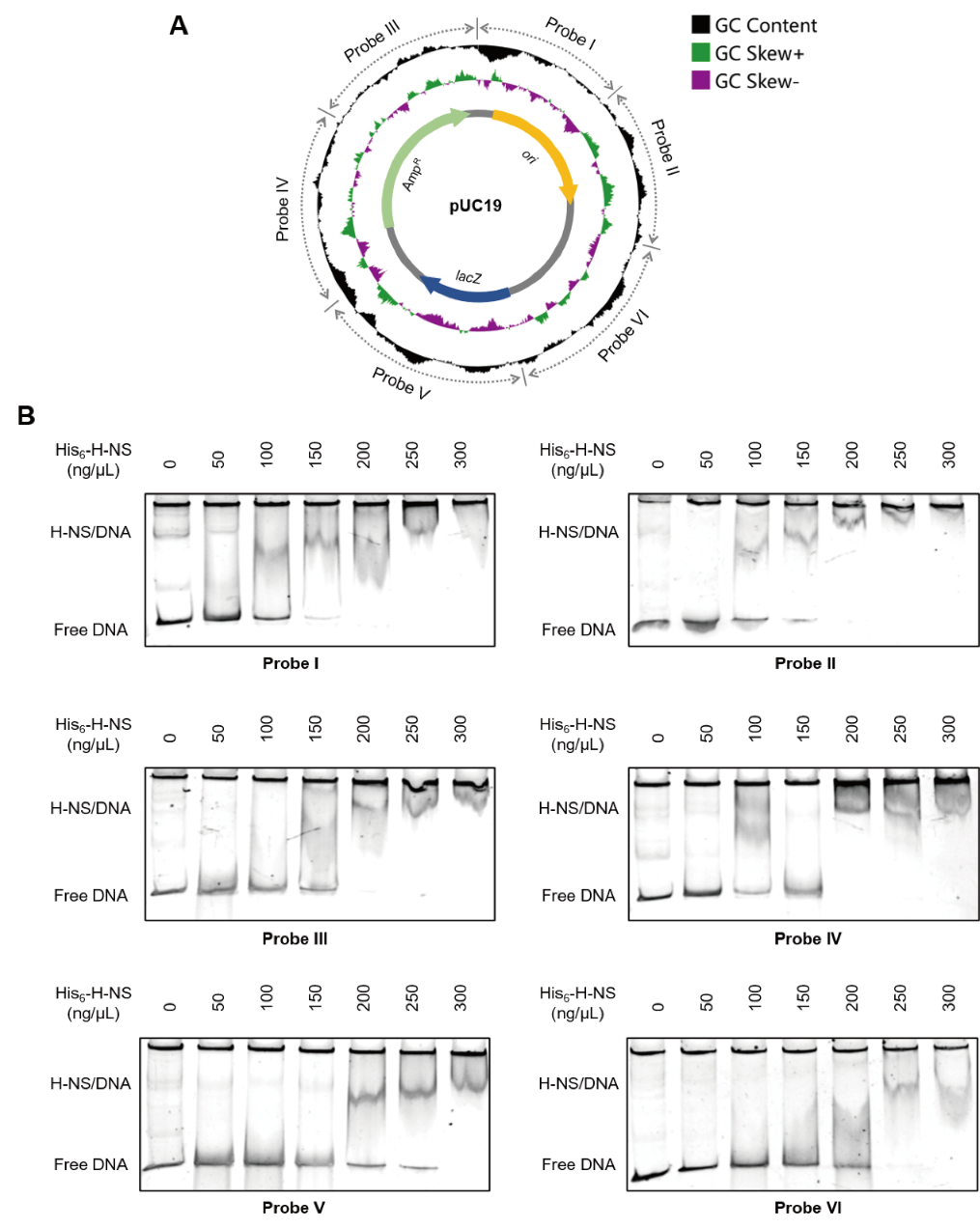


**Supplementary Figure S8.** Binding affinity of H-NS protein to different regions of pUC19. (**A**) Schematic diagram of probes derived from the pUC19 plasmid. The GC content and GC skew of pUC19 were visualized by Proksee (https://proksee.ca/). (**B**) Electrophoretic mobility shift assay with probes shown in (**A**) was performed using His_6_-H-NS. The sample was separated with 5% native polyacrylamide gels. Probe concentration: 1 ng/μL.


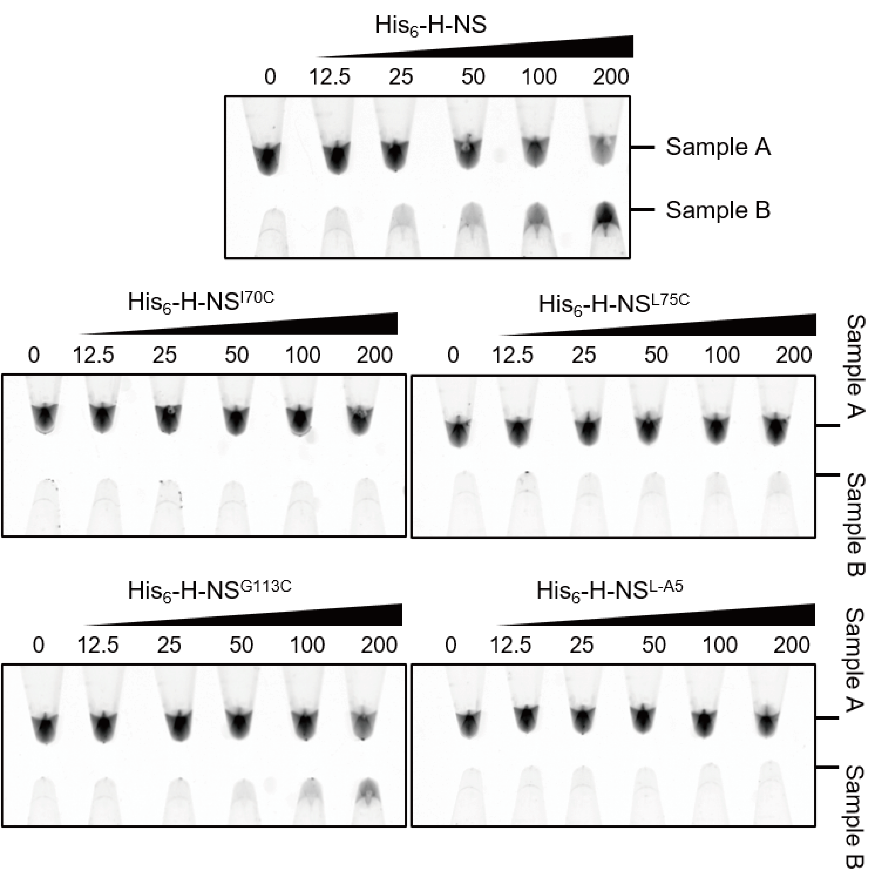


**Supplementary Figure S9.** H-NS mediates bridging between replicon and H-NS binding site. DNA-bridging assay. H-NS and its variants were tested at different concentrations for their ability to bridge Cy5-labeled fragment II probes. Free probes (supernatant, sample A) and bridged probes (eluted, sample B) were detected using an iBright 1500 scanner.


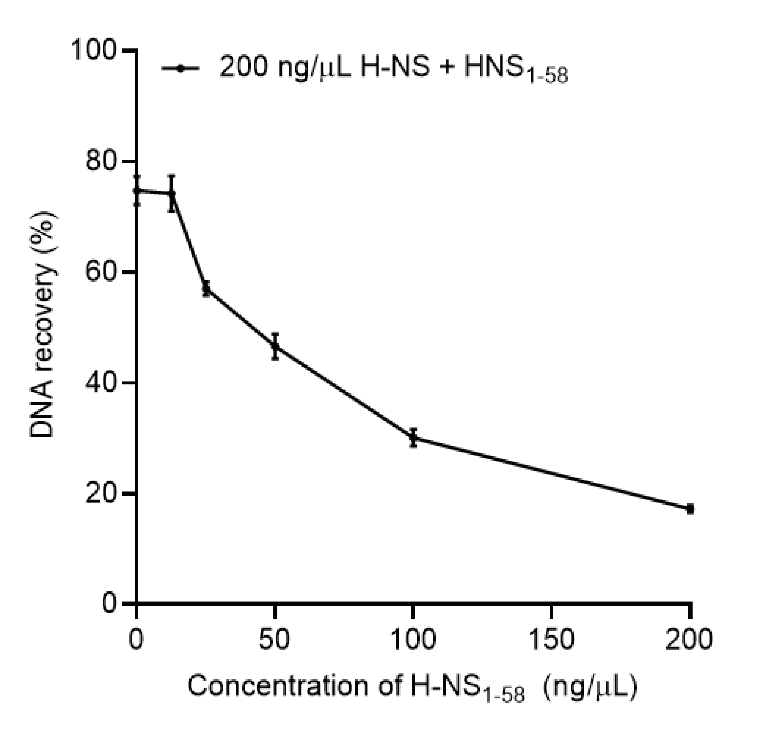


**Supplementary Figure S10.** H-NS_1-58_ variant inhibits the DNA-bridging ability of H-NS. DNA-bridging assay was conducted with biotin-labeled fragment I, Cy5-labeled fragment II, 200 ng/μL wild-type H-NS protein and different concentrations of H-NS_1-58_ variant (*n* = 2). The method for DNA bridging assay is described in Materials and methods.

**
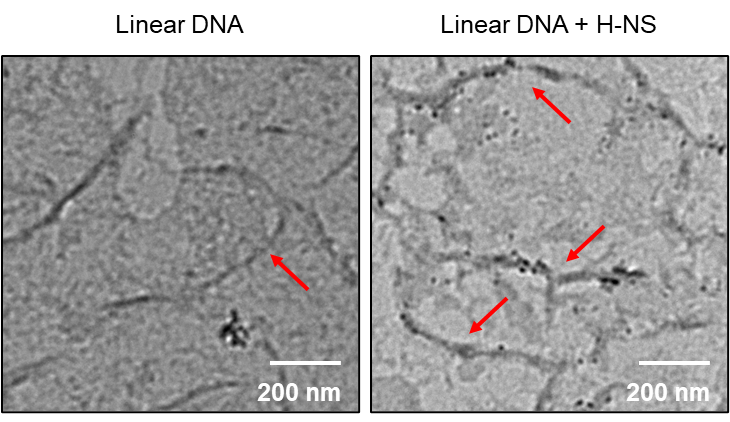
**

**Supplementary Figure S11.** TEM analysis of the structures of the naked (left) and H-NS-bound linear DNA (right), which were marked by red arrows. The linear DNA was obtained by PCR amplification from pUCsiteII with the primer pair P_112_-P_113_.

**
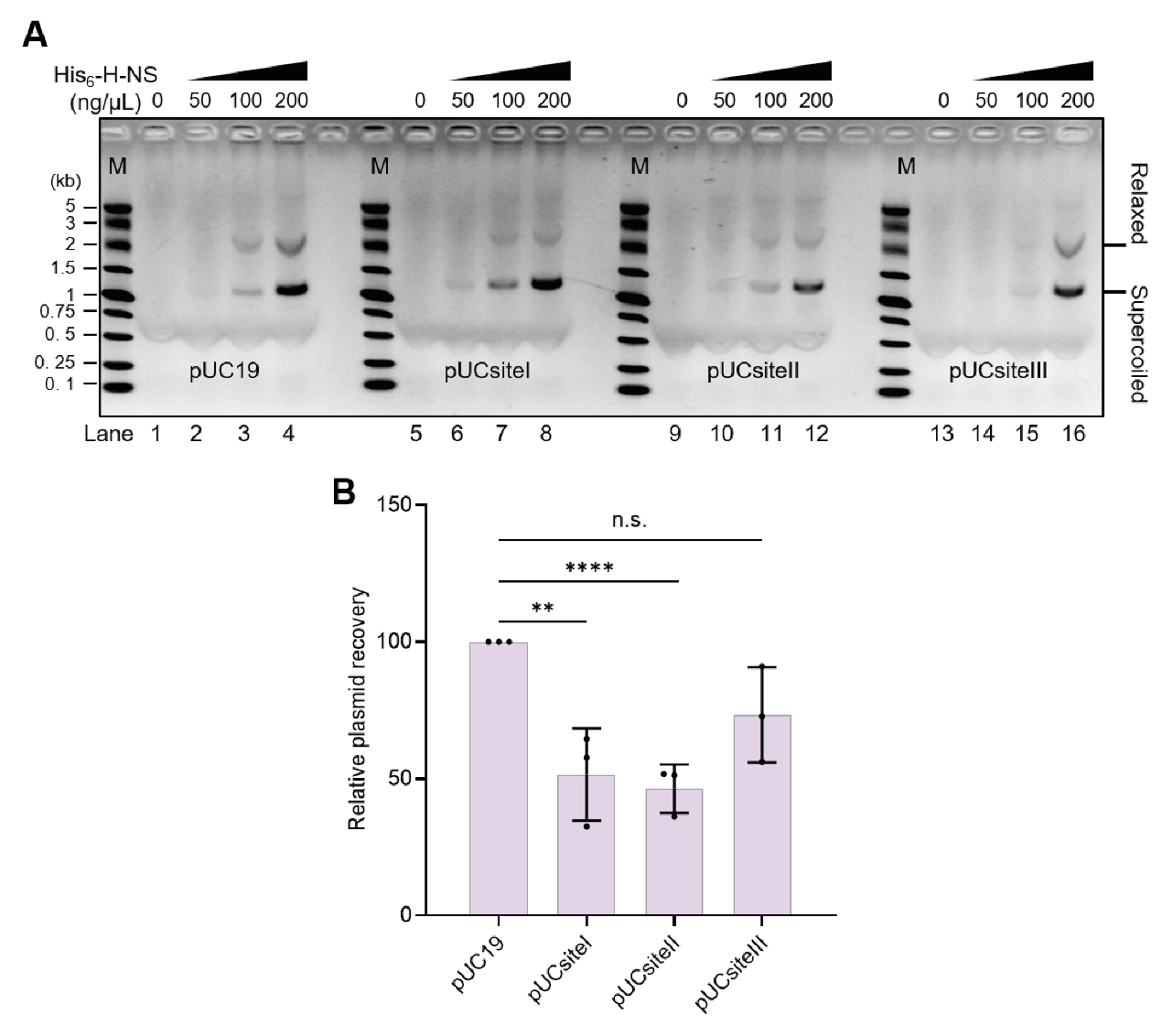
**

**Supplementary Figure S12**. (**A**) Agarose gel electrophoresis of the recovered plasmids. The bridged-plasmids were separated by 1% TAE agarose gel electrophoresis. H-NS protein concentrations: 0, 50, 100 and 200 ng/μL. M: 5000 DL marker. (**B**) The ability of H-NS (200 ng/μL) to bridge plasmids and probes was quantified for pUC19, pUCsiteI, pUCsiteII, and pUCsiteIII (*n* = 3). The amount of relaxed plasmids bridged by H-NS was quantified using ImageJ, with the bridged pUC19 plasmid set to 100%. Statistical significance was assessed using a two-tailed Student’s *t*-test (** *p* ≤ 0.01, **** *p* ≤ 0.001, n.s. *p* > 0.05). Data are shown as the mean ± SD.


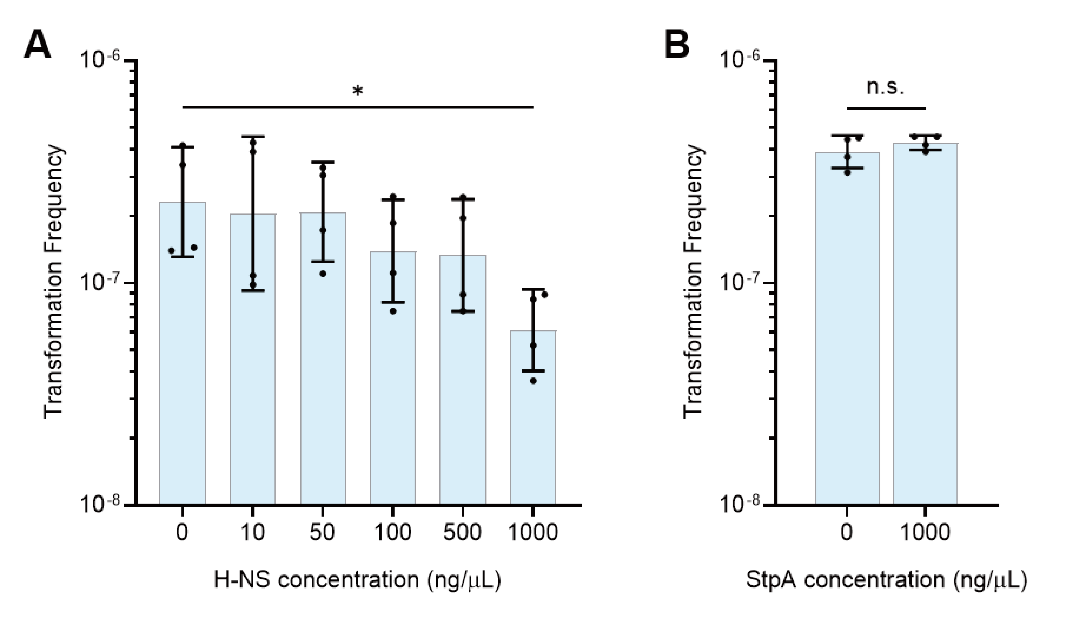


**Supplementary Figure S13.** Pre-treating plasmid with H-NS under conditions conducive to *in vitro* DNA bridging reduces transformation frequency. (**A**) Plasmid pDSRED (4 μg) was incubated with different concentrations of H-NS protein (total volume 10 μL) for 20 min at 30°C, and then was transferred into the Δ*hns* strain through natural transformation (*n* = 4). (**B**) The plasmid was incubated with StpA protein as a control (*n* = 4). The method for natural transformation is described in Materials and methods. Statistical significance was assessed using a two-tailed Student’s *t*-test (* *p* ≤ 0.05, n.s. *p* > 0.05). Data are shown as the geometric mean ± geometric SD.


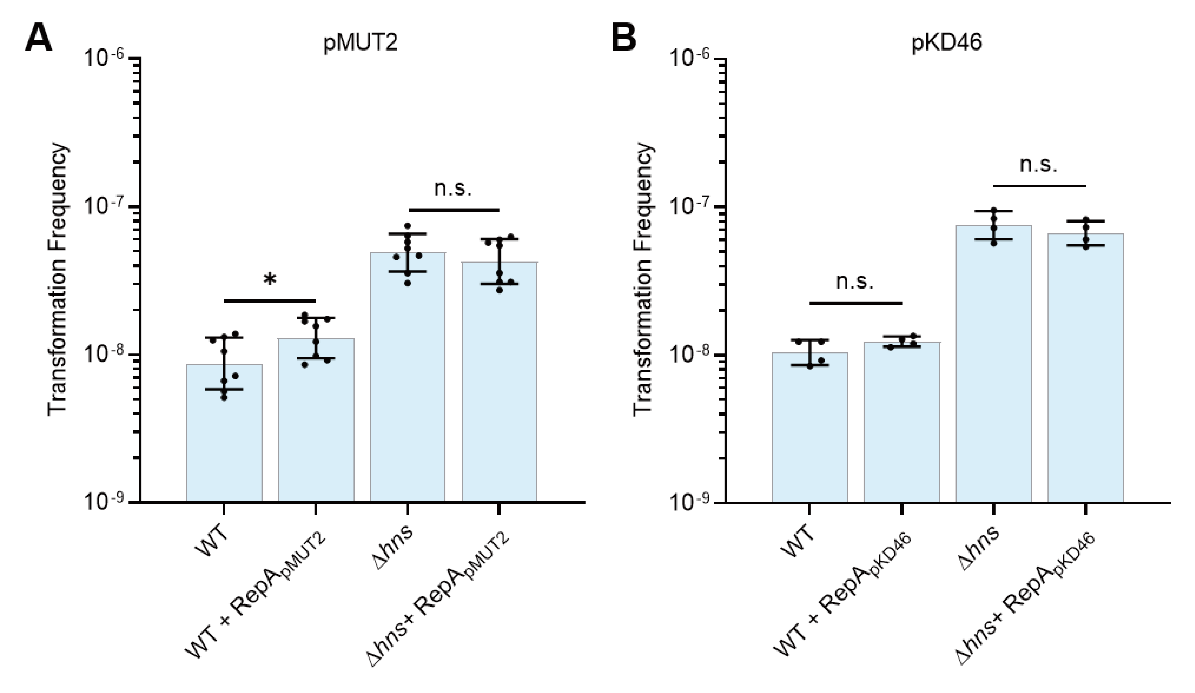


**Supplementary Figure S14.** Effect of plasmid-encoded initiator on plasmid transfer. (**A**) TFs of the WT, WT + RepA_pMUT2_, Δ*hns* and Δ*hns* + RepA_pMUT2_ strains were evaluated using ColE2 plasmid pMUT2 (*n* = 8). (**B**) TFs of the WT, WT + RepA_pKD46_, Δ*hns* and Δ*hns* + RepA_pKD46_ strains were evaluated using pSC101 plasmid pKD46 (*n* = 4). RepA_pMUT2_ and RepA_pKD46_ were constitutively expressed from the chromosome. Data are shown as the geometric mean ± geometric SD. Statistical significance was assessed using a two-tailed Student’s *t*-test (* *p* ≤ 0.05, n.s. *p* > 0.05).


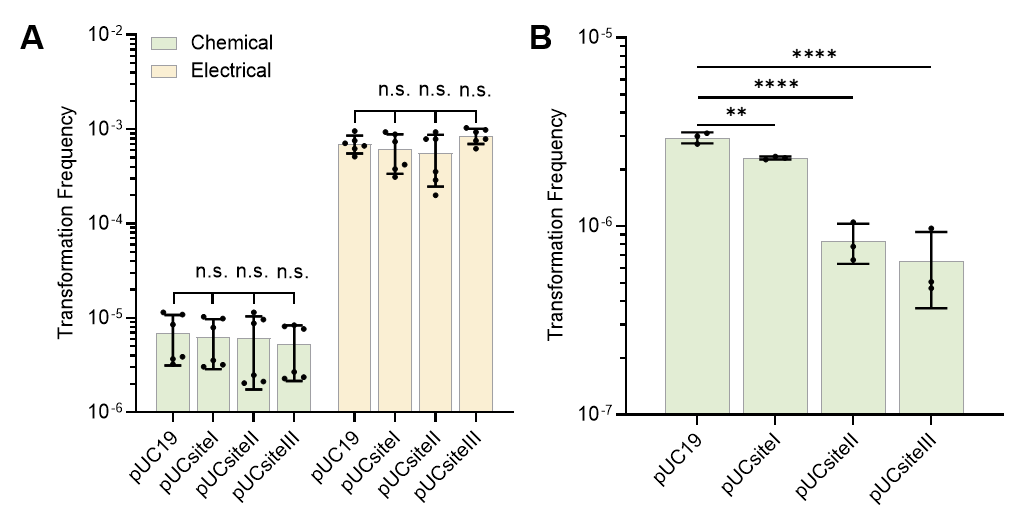


**Supplementary Figure S15.** Evaluation of plasmid transfer during artificial transformation. (**A**) Effect of H-NS binding site (HBS) position on transformation. Plasmids pUCsiteI, pUCsiteII, and pUCsiteIII were generated by placing HBS at different positions on pUC19 (refer to Figure 4C), and separately transferred into the wild-type strain by chemical transformation and electroporation methods (*n* = 6). TF was calculated as the ratio of transformants to viable cells. Statistical significance of TF was assessed using a one-way ANOVA with Dunnett’s multiple comparisons test against the pUC19 (n.s. *p* > 0.05). (**B**) TFs of the Δ*hns* strain during chemical transformation at 30°C with plasmids containing HBS. Plasmids pUC19, pUCsiteI, pUCsiteII and pUCsiteIII were transferred into Δ*hns* strain (*n* = 3). The chemically competent cells were incubated at 30°C for 30 min before heat-shocking. Statistical significance was assessed using a two-tailed Student’s *t*-test (n.s. *p* > 0.05). Data are shown as the geometric mean ± geometric SD.

**
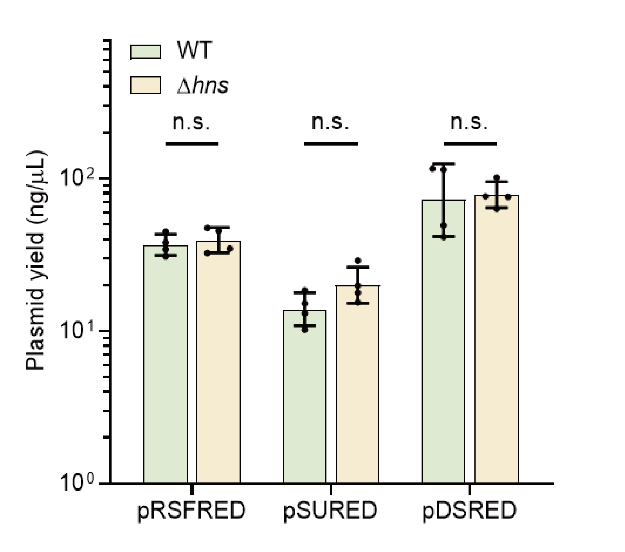
**

**Supplementary Figure S16.** Impact of H-NS on plasmid copy number. Plasmid yields of pRSFRED, pSURED, and pDSRED were quantified in the Δ*hns* mutant and its wild-type (WT) parent strain BW25113. Plasmid yield was calculated by dividing the plasmid concentration by OD_600_ (*n* = 4). Statistical significance was assessed using a two-tailed Student’s *t*-test (n.s. *p* > 0.05). Data are shown as the geometric mean ± geometric SD.

**Supplementary References**

1. Sun DC, Mao XD, Fei MY *et al.* Histone-like nucleoid-structuring protein (H-NS) paralogue StpA activates the type I-E CRISPR-Cas system against natural transformation in *Escherichia coli*. *Appl Environ Microb*, 2020, **86**: e00731-20. <https://doi.org/10.1128/AEM.00731-20>

2. Gao Y, Foo YH, Winardhi RS *et al.* Charged residues in the H-NS linker drive DNA binding and gene silencing in single cells. *Proc Natl Acad Sci USA*, 2017, **114**:12560-12565. <https://doi.org/10.1073/pnas.1716721114>

3. Datsenko KA and Wanner BL. One-step inactivation of chromosomal genes in *Escherichia coli* K-12 using PCR products. *Proc Natl Acad Sci USA*, 2000, **97**:6640-6645. <https://doi.org/10.1073/pnas.120163297>

4. Sun DC. Two different routes for double-stranded DNA transfer in natural and artificial transformation of *Escherichia coli*. *Biochem Biophys Res Commun*, 2016, **471**:213-218. <https://doi.org/10.1016/j.bbrc.2016.01.137>

5. Sun DC, Wang B, Zhu LH *et al.* Block and boost DNA transfer: Opposite roles of OmpA in natural and artificial transformation of *Escherichia coli*. *PLoS One*, 2013, **8**:e59019. <https://doi.org/10.1371/journal.pone.0059019>

6. Saltikov CW and Newman DK. Genetic identification of a respiratory arsenate reductase. *Proc Natl Acad Sci USA*, 2003, **100**:10983-10988. <https://doi.org/10.1073/pnas.1834303100>

7. Yuan PH, Xu MT, Mao CY *et al.* Dynamically regulating glucose uptake to reduce overflow metabolism with a quorum-sensing circuit for the efficient synthesis of d-pantothenic acid in *Bacillus subtilis*. *ACS Synth Biol*, 2023, **12**:2983-2995. <https://doi.org/10.1021/acssynbio.3c00315>

8. Lippa AM, Gebhardt MJ and Dove SL. H-NS-like proteins in *Pseudomonas aeruginosa* coordinately silence intragenic transcription. *Mol Microbiol*, 2021, **115**:1138-1151. <https://doi.org/10.1111/mmi.14656>

9. Yin JH, Cai JX, Yuan Z *et al.* Deletion of PBP1a/LpoA complex compromises cell envelope integrity in *Shewanella oneidensis*. *FEMS Microbiol Lett*, 2018, **365**:<https://doi.org/10.1093/femsle/fny128>.

10. Norrander J, Kempe T and Messing J. Construction of improved M13 vectors using oligodeoxynucleotide-directed mutagenesis. *Gene*, 1983, **26**:101-106. <https://doi.org/10.1016/0378-1119(83)90040-9>

11. Wu L, Wang JX, Tang P *et al.* Genetic and molecular characterization of flagellar assembly in *Shewanella oneidensis*. *PLoS One*, 2011, **6**:e21479. <https://doi.org/10.1371/journal.pone.0021479>
